# Observational evidence of salt finger in the diurnal thermocline

**DOI:** 10.1038/s41598-023-30564-5

**Published:** 2023-03-03

**Authors:** K. Ashin, M. S. Girishkumar, Eric D’Asaro, J. Jofia, V. R. Sherin, N. Sureshkumar, E. Pattabhi Ram Rao

**Affiliations:** 1 Ministry of Earth Sciences (MoES), Indian National Centre for Ocean Information Services (INCOIS), Hyderabad, Telangana 500 055 India; 2 Applied Physics Laboratory and School of Oceanography, University of Washington, Seattle, USA; 3 School of Ocean Science and Technology, Kerala University of Fisheries and Ocean Studies, Panangad, Cochin, India

**Keywords:** Ocean sciences, Physical oceanography

## Abstract

Due to strong turbulent mixing, the ocean surface boundary layer region is generally not conducive to double diffusion. However, vertical microstructure profiles observations in the northeastern Arabian Sea during May 2019 imply the formation of salt fingers in the diurnal thermocline (DT) region during the daytime. In the DT layer, conditions are favorable for salt fingering: Turner angle values are between 50 and 55° with both temperature and salinity decreasing with depth; shear-driven mixing is weak with a turbulent Reynolds number of about 30. The presence of salt fingering in the DT is confirmed by the presence of staircase-like structures with step sizes larger than the Ozmidov length and by the dissipation ratio that is larger than the mixing coefficient. The unusual daytime salinity maximum in the mixed layer that supports salt fingering is primarily due to a daytime reduction in vertical entrainment of fresh water along with minor contributions from evaporation and horizontal advection and a significant contribution from detrainment processes.

## Introduction

A better understanding of different oceanic processes leading to diapycnal mixing in the ocean's interior and boundary layer region is imperative to represent these processes in ocean models using seasonal, extended, and future climate projections. Diapycnal mixing associated with salt finger and diffusive convection form of double diffusion processes are significant, particularly in the interior oceanic region where shear-driven mixing is weak^1–9^. The diffusive convection form of double diffusion occurs when colder and fresher water overlies warmer and saltier water, typically more prominent in the polar and subpolar regions ^10,11^. On the other hand, the salt finger form of double diffusion occurs when warm and salty water overlies cold and freshwater. This process is potentially active in much of the tropics and sub-tropical oceanic regions^10,11^.

Double diffusion is typically active only in the region where shear-driven mixing is weak, but few studies have shown that it can co-exist with shear-driven mixing^1–9,12^. The general notion is that the ocean's near-surface boundary layer region is not conducive for double diffusion processes due to the persistent occurrence of strong turbulent mixing due to wind and buoyancy forces. However, turbulence is often suppressed in the boundary layer during periods of low winds and strong surface heating, opening an opportunity for double diffusion to operate.

In turbulent conditions, the values of eddy diffusivity of temperature (*K*_*T*_) are generally considered as equivalent to diapycnal diffusivity or eddy diffusivity of density (*K*_*ρ*_) estimated using Osborn^13^ turbulence model with a constant value for the mixing coefficient (*γ*_*R*f_ = 0.2)^13,14^. However, the *K*_*T*_ values in the double diffusion regime are higher than the *K*_*ρ*_ estimates based on the Osborn^13^ turbulence model^1,3,5,7–9^. Consequently, downgradient heat and salt flux due to double diffusion should be higher in the double diffusion conditions than the estimation based on Osborn^13^ turbulence model. This feature suggests that double diffusion can effectively transport heat and salt down the gradient rather than mechanical-driven mixing. Besides, the vertical density flux is down-gradient in the mechanical-driven turbulence case, while it is up-gradient in the double diffusion processes. Hence, double diffusion mixing leads to an increase in the water column’s stratification compared to shear-driven turbulence, in which stratification decreases as a result of mixing^6,9^.

Past studies have speculated that the double diffusion processes can occur in the near-surface layer under favorable conditions^15,16^. The existence of diffusive convection in the upper 20 m of the water column with a weak staircase after an intense rain event was reported in a study based on microstructure-based observation^4^. However, the studies on the existence of double diffusion processes in the oceanic region’s near-surface layer using microstructure data are limited.

**Figure 1:**
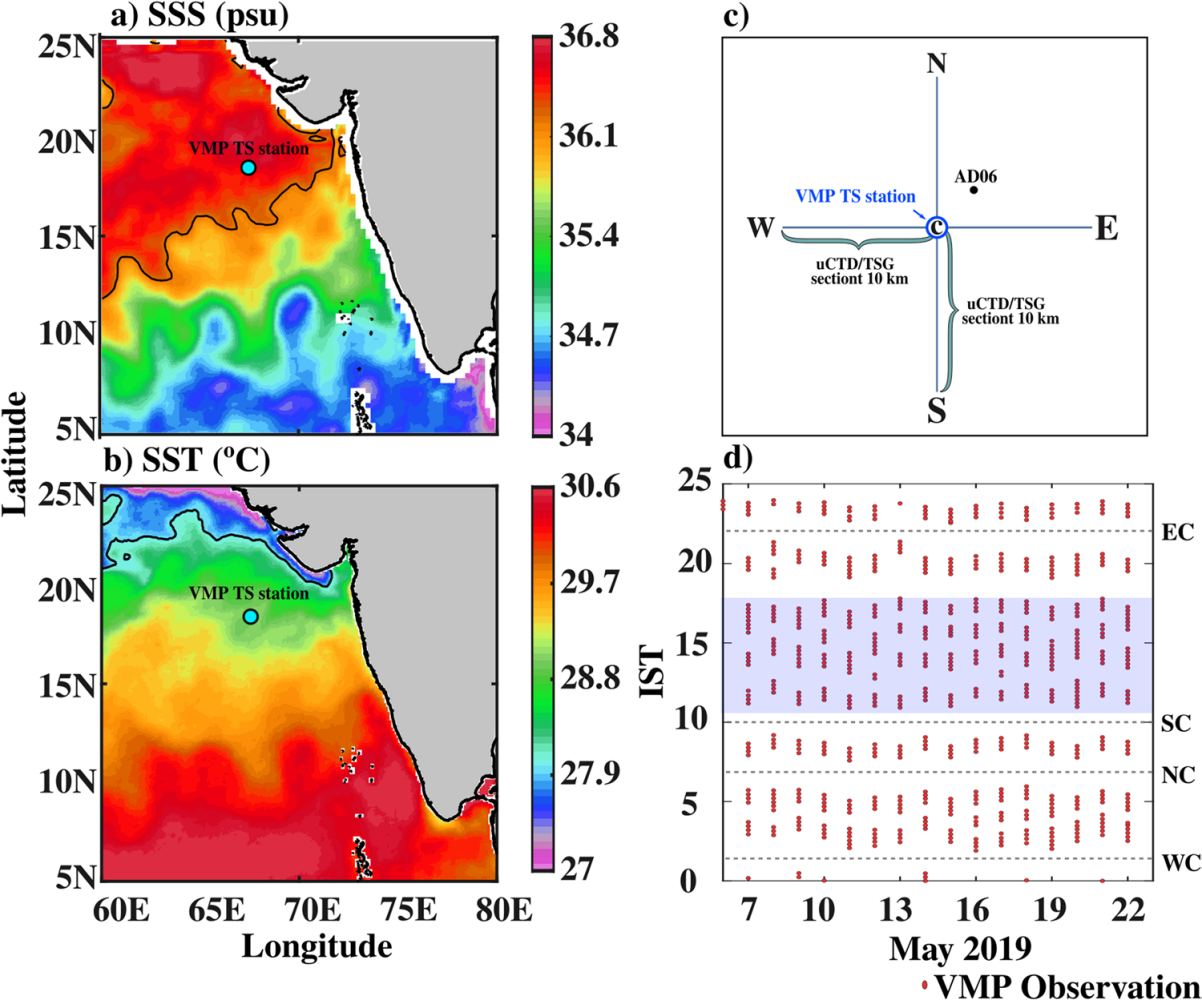
Monthly average of satellite based (**a**) sea surface salinity (psu) and (**b**) SST (°C). (**c**) The schematic of the zonal (east (E)-west (W)) and meridional (north (N)-south (S)) uCTD/TSG transects (blue lines) at the VMP time-series station at (18.4° N and 67.4° E; blue circle; marked as ‘C’). (**d**) VMP data availability time during each day (red dots). In panel (**c**) the black filled circle represents the AD06 mooring location. In panel (**d**), the approximate time of the day when uCTD/TSG transects are carried out is marked as black horizontal dotted lines (0700 IST—N–C; 1000 IST—S–C; 2200 IST—E–C; 2500 IST—W–C). In panels (**a**) and (**b**), the VMP time-series station is marked in the cyan-filled circle. The black contours in (**a**) and (**b**) are 36.2 psu and 28 °C, respectively. The light blue shade in panel (c) represents the period when uninterrupted VMP observations were carried out between 1000 and 1900 IST.

In this study, we will test for the presence of salt fingering in the surface boundary layer region in the northeastern Arabian Sea (NEAS) at 18.4°N and 67.4°E during spring using 16-day (7–22 May 2019) time series of microstructure measurements of temperature and shear, along with fine-scale measurements of temperature and salinity as described in the methods (Fig. 1; *Materials and Methods*). These measurements will be used to compute several salt finger diagnostic quantities as described below. *Tu*, the Turner Angle (*Materials and Methods: Salt finger diagnostics*), and its values between 45° and 90° indicate that vertical temperature and salinity gradients are favorable for salt fingering, and it is considered as strong for 72° ≤ Tu ≤ 90° and weaker for 45° ≤ Tu < 72°^6^. The presence of a meter-scale staircase-like structure of temperature and salinity is a strong indicator of salt fingering, and such structures are detected with an algorithm^2,4^ (*Materials and Methods: salt finger diagnostics*). The higher values of dissipation ratio (*γ*_*χε*_) compared to *γ*_*Rf*_ (0.2) is an important feature to differentiate the double diffusion regime from turbulent conditions^1,3,5,7–9^ and it is computed using the concurrent availability of turbulent kinetic energy dissipation rate (*ε*; *Materials and Methods: salt finger diagnostics*) derived from shear microstructure data and thermal variance dissipation rates* (χ; Materials and Methods: salt finger diagnostics*) estimated from temperature microstructure. Finally, the sub-daily variability of mixed layer (ML) salinity in the study region is evaluated using the ML salt budget equation (*Materials and Methods: ML salinity budget*).

## Results

### The diurnal cycle of temperature and salinity

**Figure 2:**
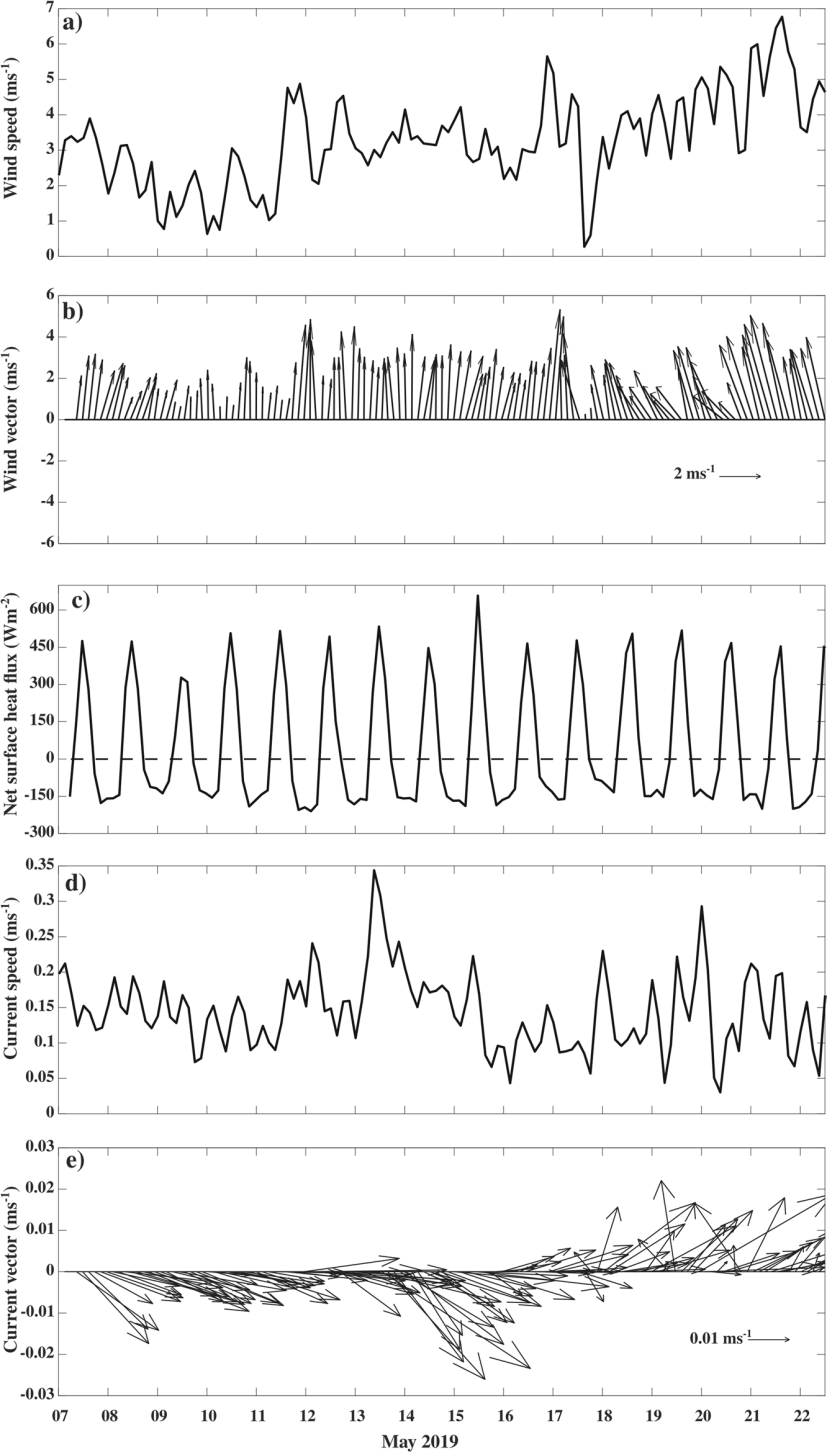
Temporal evolution of (**a**) wind speed (m s^−1^), (**b**) wind vectors (m s^−1^), (**c**) net surface heat flux (*NHF*; Wm^−2^), (**d**) current speed (ms^−1^) and (**e**) current vectors (m s^−1^) during 07–22 May 2019 at VMP time-series station (18.4° N and 67.4° E) derived from AD06 mooring. In panel (**c**) the dashed horizontal line represents *NHF* magnitude of zero. Time in IST hours.

Weak winds (< 6 m s^−1^) persisted throughout the observation period in the NEAS (Fig. 2a,b). The diurnal range of net surface heat flux (NHF) shows a magnitude of ~ 800 Wm^−2^ with a maximum heat gain by the ocean during the afternoon (~ 450 Wm^−2^) and maximum heat loss from the ocean during the night (~ − 150 Wm^−2^) (Fig. 2c).

**Figure 3:**
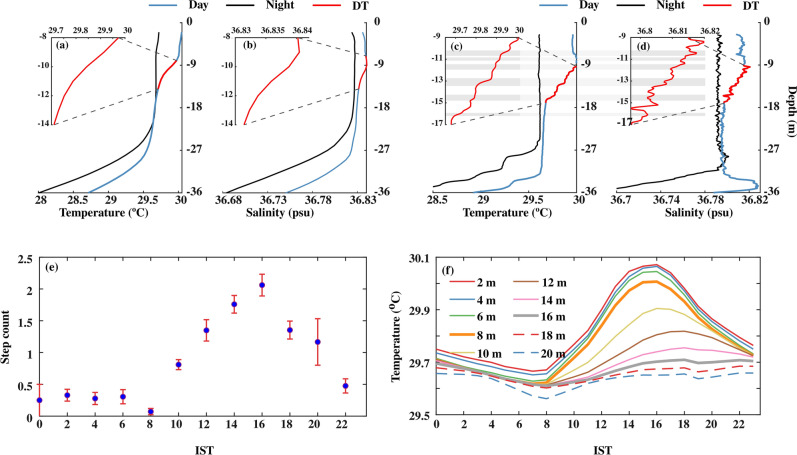
(**a** and **b**) The composite (07–22 May 2019) average and (**c** and **d**) a typical example of the vertical profile of (**a** and **c**) temperature (°C) and (**b** and **d**) salinity (psu) during the afternoon (blue; 1500 IST) and night (black; 0300 IST) at the VMP time-series station (18.4° N and 67.4° E). The composite of sub-daily evolution of (**e**) average steps per profile between 2 and 20 m in every two-hour bins in the diurnal thermocline region and (**f**) average temperature (°C) at different depths demonstrates the existence of DT. The red profiles in the upper left side of (**a**), (**b**), (**c**), and (**d**) are zoomed versions of black temperature and salinity profiles in the DT region. The grey horizontal shading in panels (**c**) and (**d**) represents the staircase-like structure identified based on temperature criteria. We presented the case with the maximum number of steps observed during the entire time series observation in panel (**c**). However, the number of steps per profile is smaller than in this typical case, and its composite evolution during the time series observation is presented in panel (**e**).

**Figure 4:**
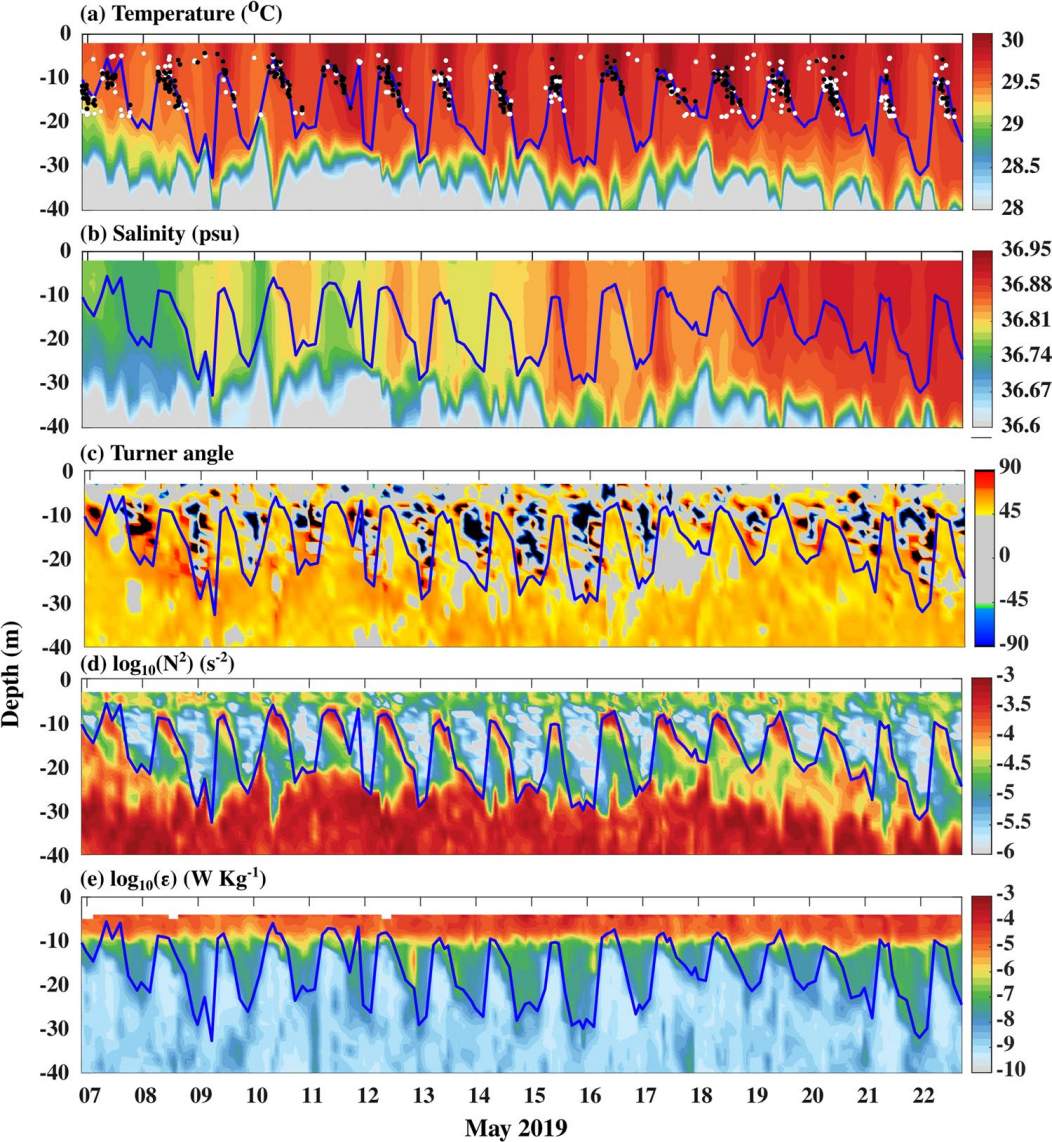
The depth time section of (**a**) temperature (°C), (**b**) salinity (psu), (**c**) Turner angle (*Tu*, °), (**d**) *N*^2^ (s^−2^), and (**e**) log_10_(ε) (W kg^−1^) during 07–22 May 2019 at VMP time-series station (18.4° N and 67.4° E). The dots in panel (**a**) represent the center point of the interface in the staircase structure detected in the DT region. The staircase structure with an interface after a layer is marked as white dots, and the layer above and below the interface is marked as black dots. The blue line in panels represents MLD.

**Figure 5:**
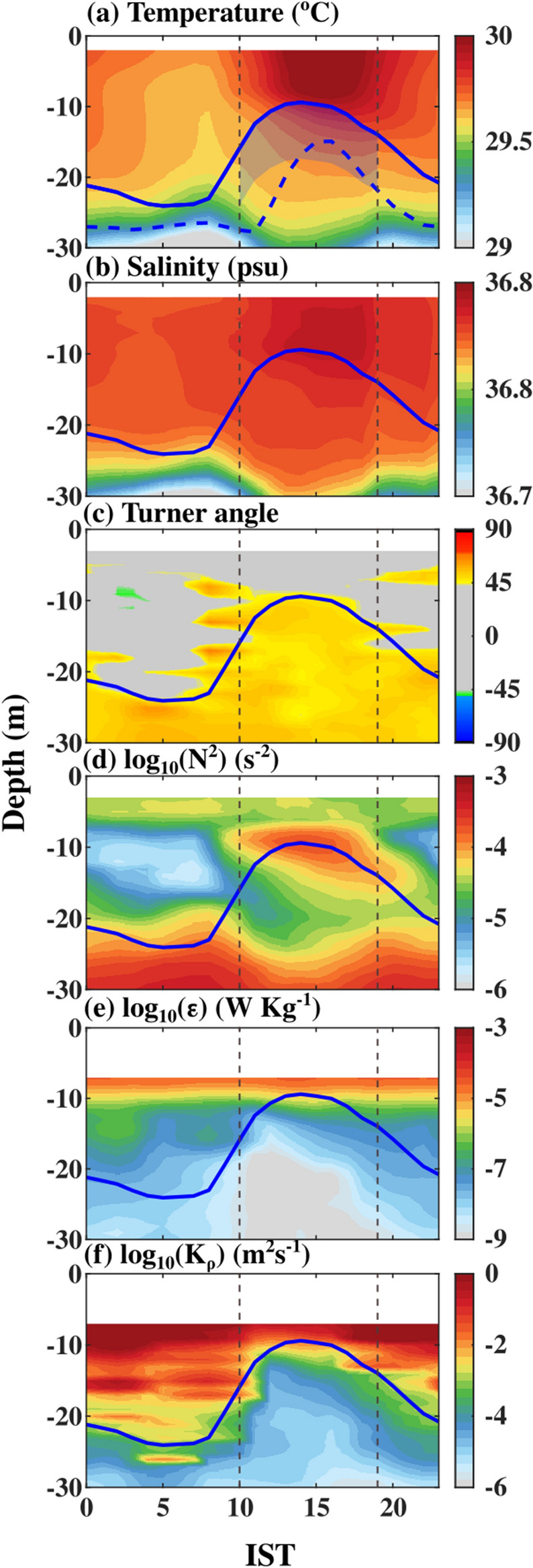
The composite (07–22 May 2019) of sub-daily evolution of (**a**) temperature (°C), (**b**) salinity (psu), (**c**) Turner angle (*Tu*, °), (**d**) *N*^2^ (s^−2^), (**e**) log_10_(ε) (W kg^−1^) and (**f**) log_10_(*K*_*ρ*_) (m^2^ s^−1^). The blue line in the panels represents the MLD estimated using 0.1 °C temperature criterion. The blue dotted line in panel (**a**) represents the depth where the temperature is 0.3 °C lower than the surface value. The dotted vertical lines in the panels represent the time period used to construct a composite of parameters in the DT region (marked as grey shading in panel (**a**) in Figs. 7 and 8. Time in IST hours.

**Figure 6:**
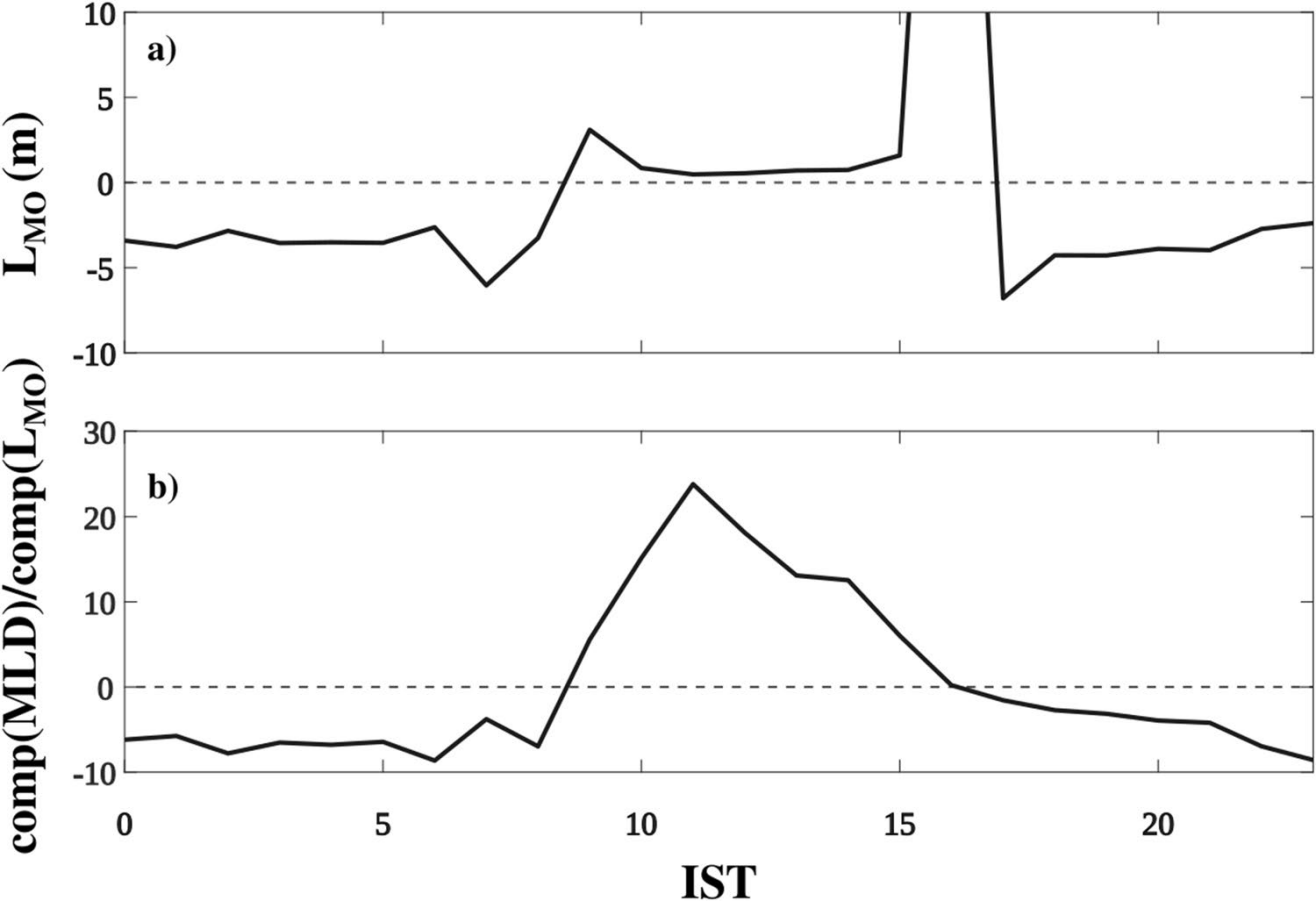
Composite of sub-daily evolution of (**a**) Monin–Obukhov length (*L*_*MO*_, m), and (**b**) the ratio between MLD and Monin–Obukhov length ($${L}_{MO})$$ ($$\frac{MLD}{{L}_{MO}}$$). In the bottom panel, the sub-daily composite of MLD and L_MO_ are used to estimate the sub-daily evolution of $$\frac{MLD}{{L}_{MO}}$$. The composite of sub-daily evolution of buoyancy flux (*B*_*f*_ × 10^−7^; m^2^ s^−1^) and frictional velocity (U^*^, ms^−1^) are presented in Fig. S9a,b, respectively. The MLD was estimated using 0.1 °C temperature criterion. Time in IST hours.

The depth-time section of temperature and its mean diurnal evolution during spring 2019 shows the existence of warm (~ 30 °C) water over cold (~ 28 °C) water in the upper 30 m (Figs. 3a, c, 4a, and 5a). Similarly, the vertical distribution of salinity in the upper 30 m water column shows slightly high salinity water (~ 36.95 psu) over relatively low salinity water (~ 36.6 psu) (Figs. 3b, d, 4b, and 5b). In response to the strong diurnal range of NHF under weak wind speed conditions, the thermohaline structure has a substantial diurnal variation in the upper 20 m of the water column with daytime warming and salinification with a peak value around 1400 IST-1600 IST and cooling and freshening during the night with a minimum value before the dawn (Fig. 5a, b). The temperature and salinity profiles thus show a distinct vertical structure between daytime and night (Fig. 3a, b). During the night, wind-induced vertical mixing and convective overturn due to NHF loss from the sea surface (Fig. 2a, c) lead to the formation of a well-defined isothermal and isohaline layer in the upper 25–30 m of the water column (Figs. 3a–d, 4a, b and 5a, b). The ratio between ML depth (MLD) and Monin–Obukhov length ($L_{MO}$) shows a value much less than -1 *(Materials and Methods: Monin–Obukhov length)*, indicating the importance of convective overturn during ML deepening during the night (Fig. 6a, b). During the daytime, enhancement of the NHF due to the increased solar radiation in the presence of weak wind leads to the formation of a shallow warm daytime ML (Figs. 2a–d, 3a, 4a, and 5a).

The diurnal thermocline (DT), a transition layer between the base of warm daytime ML and nocturnal ML**,** is apparent in the vertical temperature profile when the surface warms above the background temperature of the nocturnal ML (Figs. 3a, c, 4a, and 5a). This temperature stratification is evident as higher values of the Brunt-Väisälä frequency** (N**^*2*^ > 10^−3^ s^−2^) around 10–20 m depth between 1000 and 1900 IST compared to its values in a similar depth during the night (~ 10^−6^ s^−2^) (Figs. 4d and 5d). Importantly, the salinity decreases with depth with a gradient of ~ 0.0026 psu m^−1^ in the DT region during the daytime (Figs. 3b, 4b, and 5b).

The upward increase in temperature in the DT is commonly observed and expected, but the upward increase in salinity is unusual and makes the DT susceptible to salt-fingering form of double diffusion. We now consider the other indicators for the presence of such double diffusion.

### Salt fingering

The near-surface values of *ε* are around ~ 10^−5^ W Kg^−1^ throughout the day, and these characteristics are comparable with previous studies^17,18^ (Figs. 4e and 5e). During the night, the higher values of the *ε* (> 10^−7^ W Kg^−1^) are observed in the upper 20 m of the water column within the nighttime ML (Figs. 4e and 5e). The enhancement of near-surface thermal stratification during the daytime inhibits the vertical transfer of momentum from the wind, and it traps in the shallow near-surface layer. These characteristics reduce the magnitude of *ε* (~ 10^−9^ W Kg^−1^) in the DT region around 10–20 m during the daytime, which is approximately two orders lower than the values in a similar depth level during the night (Figs. 4e and 5e). Our microstructure analysis shows the mean and standard deviation value of *K*_*ρ*_ (estimated by fitting the data through a log-normal distribution; *Materials and Methods*) during the peak phase of DT (averaged between 1000 and1900 IST and from MLD + 2 m to MLD + 8 m) is approximately 3.4 × 10^−5^ m^2^ s^−1^ and 7.1 × 10^−5^ m^2^ s^−1^, respectively, primarily due to suppression of the downward penetration of turbulence associated with enhancement of daytime stratification in the near-surface layer by solar radiation (Fig. 5f).

*Re*_*b*_, the buoyancy Reynolds number (*Materials and Methods*) measures the intensity of turbulent mixing in the water column. Salt fingering is suppressed for *Re*_*b*_ > 200^5^. The frequency histogram of *Re*_*b*_ values in the DT regions between 1000 and 1900 IST showed that very few data points (11%) fall above 200 and approximately 80% of values fall below 100 with a median (standard deviation) of 33.3 (235) (Fig. S1). Thus, the DT is a region of low turbulence.

**Figure 7:**
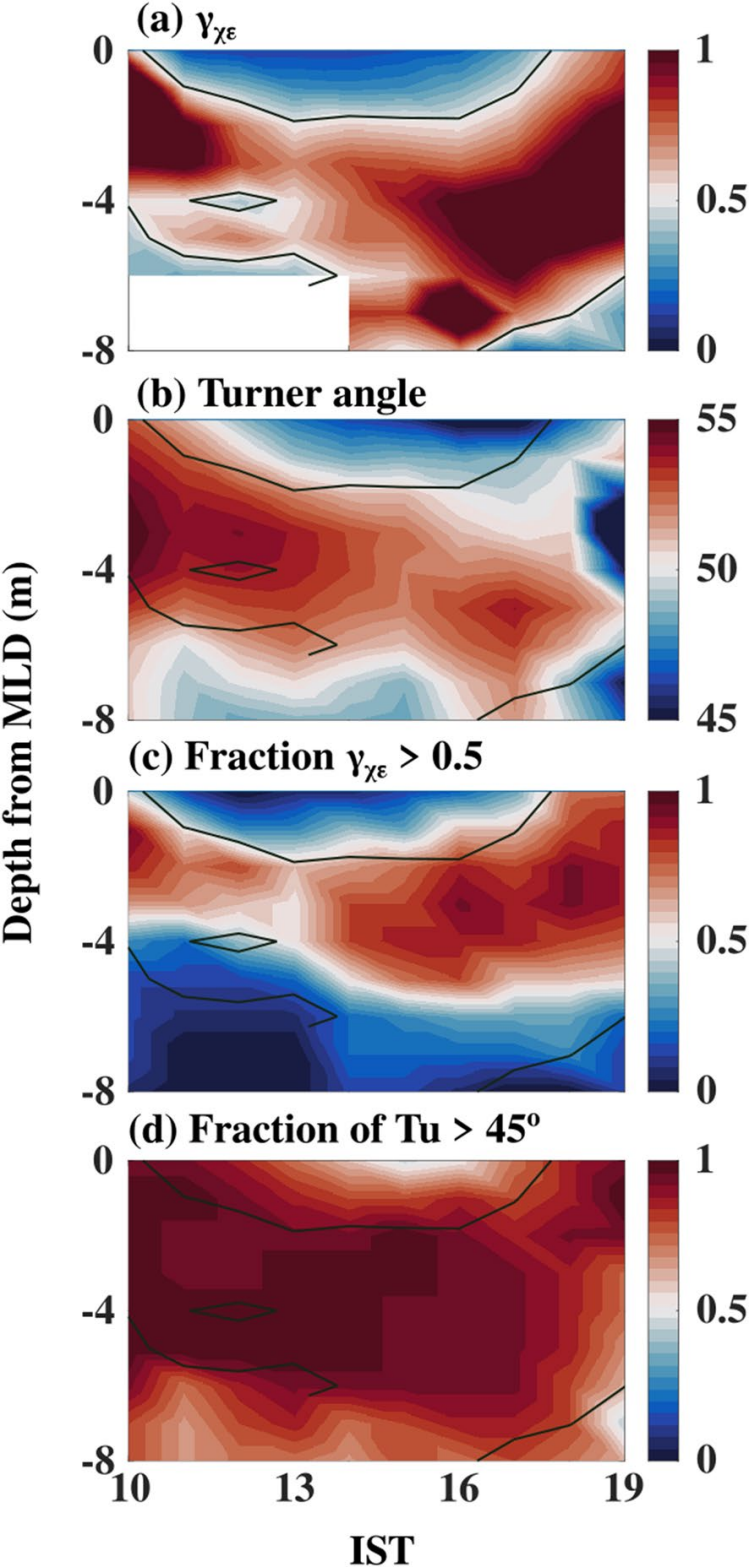
The composite (07–22 May 2019) of sub-daily evolution of (**a**) *γ*_*χε*_ (**b**) Turner angle (*Tu*,°) (**c**) fraction of γ_χε_ values greater than 0.5 and, (**d**) fraction of Turner angle (*Tu*, °) values greater than 45° between 1000 and 1900 IST in the DT region. Black contours in the panel (**a**) to (**b**) represent mixing coefficient (γ_χε_) values equal to 0.5. The y-axis in the panels represents the depth from the MLD to MLD+ 8 m. The MLD was estimated using the 0.1 °C temperature criterion. Time in IST hours.

The Turner angle shows the values in the DT region fall between 45° and 90°, of which most of the values exist between 50° and 55° (Figs. 4c, 5c, and 7b). The fraction of observations in each depth bin that is conducive for the salt finger (45° < Tu < 90°) in the DT region between 1000 and 1900 IST shows that approximately 90% of observation in these regions is conducive for the salt finger form of double diffusion (Fig. 7d). The Turner angles values are between 50° and 55°, and weak shear-driven mixing suggests the existence of salt fingers in the DT region of NEAS.

### Staircase structure

A typical temperature profile during the peak diurnal warming shows a staircase-like structure in the DT region (Figs. 2c). We consider a staircase structure only if the interface occurs immediately after a layer. Consistent with the observations based on a single profile, our analysis shows numerous staircases in the DT at this time (Fig. 4a; white and black dots). The staircase structure is also apparent in the near-surface region during the nighttime, though it is sporadic (Figs. 3e and 4a). The total number of staircase structure count progressively increases from 1000 IST and reaches a maximum value (2 steps per profile) around 1600 IST (Figs. 3e). They then decrease afterward, reaching a minimum value around 2400 IST, and do not show any significant variability until dawn (0600 IST) (Figs. 3e and 4a). A robust temporal correspondence between the strength of the DT and staircase-like structure counts is evident, such as the maximum strength of DT coincides with the period of frequent formation of steps during the afternoon (Figs. 3e and 4a).

**Figure 8:**
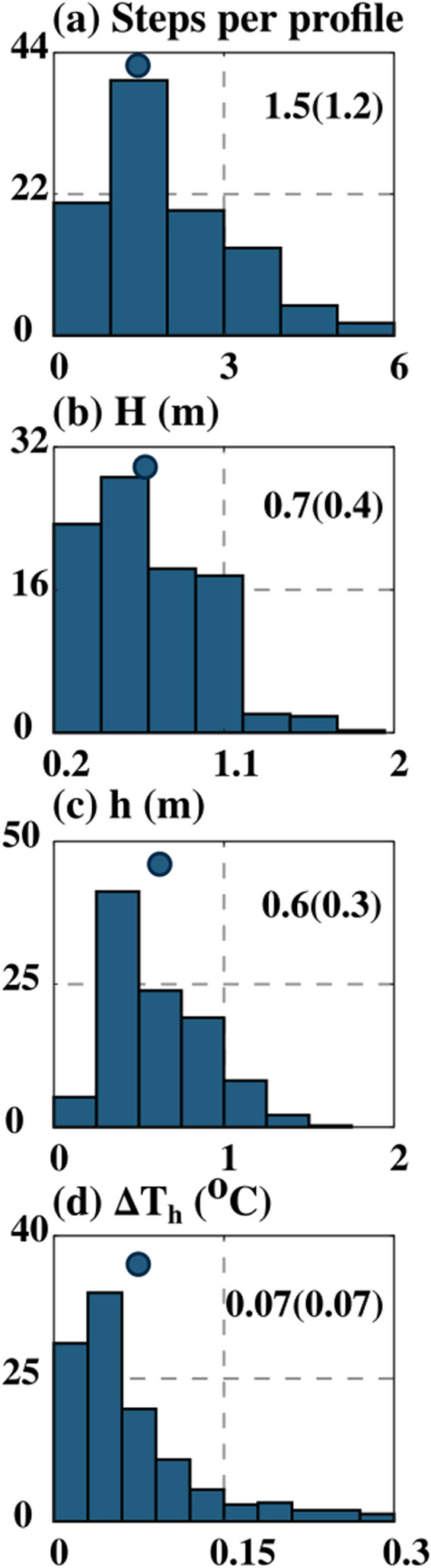
Frequency distribution (%) of properties of staircase statistics between 1000 and 1900 LST in the DT region at VMP time-series station (18.4° N and 67.4° E). (**a**) the number of steps per profile, (**b**) layer height (*H*, m) (**c**) interface height (*h*, m) between two layers, and (**d**) temperature difference (Δ*T*_*h*_, °C) across the interface. The circle inside the panel represents the mean value of each parameter. The number inside the panel represents the mean and one standard deviation (within the bracket).

The statistics of staircase structure properties in the DT region (between the base of ML to 20 m) between 1000 and 1900 IST are presented in Fig. 8. The mean (standard deviation) steps per profile are around 1.5 (1.2), and approximately 20% of profiles have more than two steps per profile (Fig. 8a). The mean (standard deviation) thickness of layer (*H*) and interface (*h*) is 0.7 (0.4) m and 0.6 (0.3) m, respectively, and approximately 30% of the layer has a thickness greater than 0.7 m, and approximately 70% interface in the staircase has a thickness less than 0.6 m (Fig. 8b,c). In addition, 50% of the layers have a height greater than the interface. The mean temperature gradient across the interface (*∆T*_*h*_) is around 0.07 °C, and approximately 25% of these magnitudes are higher than its mean value (Fig. 8d). It is also found that 63% of the staircase structure formed between 1000 and 1900 IST has a layer above and below the interface (Fig. 4a; black dots).

These statistics of staircase structure properties suggest that staircase-like structure detected in the DT region is not well-defined compared to structures observed in the oceanic region (e.g., Artic, North Atlatic), which are consistently favorable for a strong-salt finger^6,19^. Previous studies have shown that double diffusion can be active between two intermittent turbulent regimes^1^*.* Besides, the formation of staircase-like structures, though not well defined in nature, was also reported within an hour when the conditions were favorable for double diffusion^4^. Studies based on a numerical model experiment showed that the typical time scale of the formation of approximately 1–2 m spaced well-defined thermohaline staircase structure in the salt finger regime is about one day^20^. However, the salt finger favorable regime associated with the DT region in the NEAS persists less than this typical time scale and may not be sufficient to form an organized, well-defined staircase-like structure. It is worth pointing out that, the laboratory experiment using salt and sugar (equivalent to a salt finger favorable regime) demonstrated that a new staircase (layer and interface) can be formed within 2 h^21^. In addition, it is also found that the mean layer height (0.7 m; standard deviation 0.30 m) detected in the DT region is approximately a factor of four higher than the Ozmidov turbulence length scale (0.15 m; standard deviation 0.13 m) (Figs. S9 and 8b). These characteristics suggest the staircase-like structure is higher than the mean largest turbulence length scale estimated in the DT region.

In summary, the clustering of staircase-like structures in the DT region further supports the existence of salt fingers here.

### Dissipation ratio (***γ***_***χε***_)

In the double diffusion processes, *γ*_*χε*_ values are generally higher compared to *γ*_*Rf*_ (0.2)^1,3,5,8,9^. The composite evolution of *γ*_*χε*_ in the DT region shows that these values are generally higher than 0.5, with a mean value of 0.65 (standard deviation 0.6) (Fig. 7a). The fraction *γ*_*χε*_ observations higher than 0.5 in each depth bin in the DT region shows that approximately 90% of the data point has values higher than 0.5 (Fig. 7c). Besides, an alternate form of dissipation ratio (*γ*_*Rρ*_) estimated using the density ratio (*R*_*ρ*_), and flux ratio (*r;* a function of *R*_*ρ*_) in the DT region shows a comparable magnitude (0.46; standard deviation 0.04) with *γ*_*χε*_ (0.65; standard deviation 0.6). A reasonable agreement between *γ*_*Rρ*_ and *γ*_*χε*_ in the DT region suggests that our microstructure-based estimates are reasonable despite uncertainties associated with the estimation of turbulence parameters as mentioned in the materials and method (*Materials and Methods: salt finger diagnostics*).

The presence of a staircase structure, along with the higher value of *γ*_*χε*_ (> 0.5) and *γ*_*Rρ*_ (~ 0.5) compared to *γ*_*Rf*_ (0.2), provides additional evidence to support our hypothesis for the existence of salt finger in the DT region of the NEAS.

### Diurnal variability of ML salt budget

**Figure 9:**
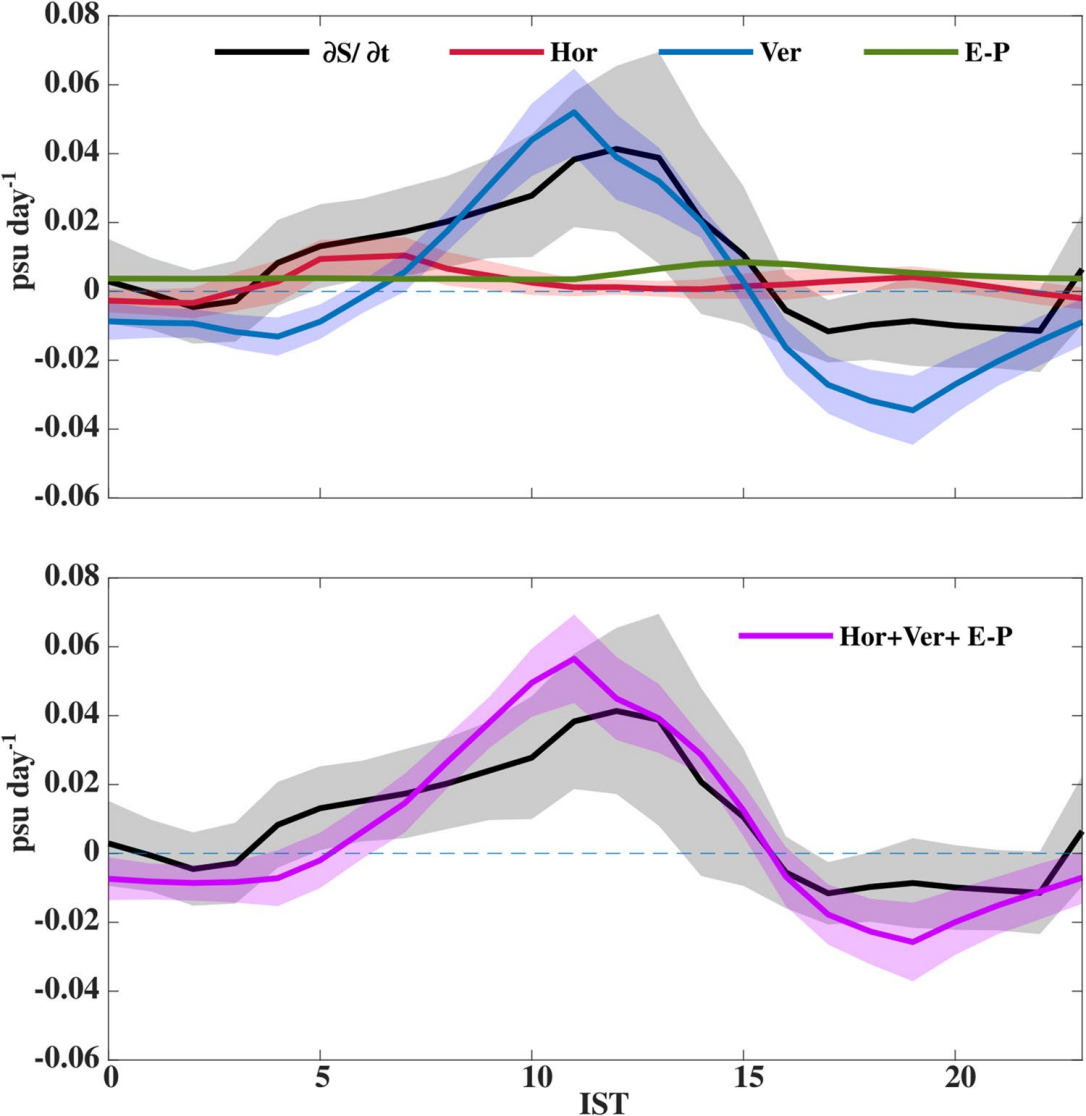
The composite (07–22 May 2019) of sub-daily evolution of different terms in the ML salt budget analysis (psu day^−1^): (**a**) rate of change of ML salinity ($\frac{\partial S}{\partial t};$ black), horizontal advection (Hor; red), vertical processes (Ver; blue), and local freshwater flux (E-P; green). and (**b**) rate of change of ML salinity ($\frac{\partial S}{\partial t};$ black), and the sum of horizontal advection, vertical processes and local freshwater flux (Hor + Ver + E-P; pink). The shading represents one standard error of the mean, and it is estimated based on the deviations of data from the mean in each 1-h bin using the bootstrap method. The dashed horizontal line represents the zero value of salinity tendency. Time in IST hours.

The formation of a shallow warm daytime ML owing to the enhancement of solar radiation, particularly during the low wind speed conditions (< 6 m s^−1^) as apparent during May 2019 in the Arabian Sea, is well documented in the earlier literature^22–24^. However, the enhancement of near-surface salinity during the daytime is quite unusual^25,26^, and the plausible reason for the sub-daily variability of near-surface salinity in the NEAS during spring is examined using ML salt budget analysis. The primary objective here is to quantify the relative importance of various terms responsible for near-surface salinification during the daytime. For that purpose, we evaluated the sub-daily composite of different terms of an upper-ocean salt budget (Fig. 9), specifically the budget for the near-surface region consisting of ML and DT (0.3 °C DT criterion, *Materials and methods: ML salinity budget*) and it is called the “ML salinity budget” for the sake of brevity. This budget closes reasonably well, which gives us the confidence to quantify the relative importance of various terms that determine the enhancement of near-surface salinity during the daytime (Fig. 9b).

The ML salinity shows salinification and freshening tendency during the daytime and nighttime, respectively, though the magnitude of salinification tendency during the daytime (~ 0.04 psu day^−1^) is approximately a factor four higher than the night (~ 0.01 psu day^−1^) (Fig. 9a).

The surface freshwater flux is primarily determined by evaporation, as no precipitation events were observed. The sub-daily variability of evaporation shows apparent diurnal variability with maximum value during the afternoon (~ 0.009 psu day^−1^) when the maximum magnitude of ML salinification occurs and minimum during the night (~ 0.004 psu day^−1^) (Figs. 9a and S3). The enhancement of near-surface specific humidity due to increasing sea surface temperature (SST) (specific humidity at the sea surface is defined as its saturation value with respect to SST) and reduction in air specific humidity due to entrainment of dry planetary boundary layer into the surface layer owing to the growth of atmospheric ML might be the plausible explanation for the increased evaporation during the daytime^27,28^ (Figs. 3f and S3). However, most importantly, the large amplitude of the increasing trend of ML salinity (~ 0.04 psu day^−1^) during the daytime cannot be explained by the evaporation term (~ 0.009 psu day^−1^) alone.

The horizontal advection shows a positive tendency throughout the day (~ 0.001–0.01 psu day^−1^) except a negative tendency (~ − 0.003 psu day^−1^) during the short period between 2400 and 0500 IST (Figs. 9a and S4). The southeastward transport of relatively high saline water from the north to the time-series station through prevailing southeastward current (Figs. 1a, 2e, and S5) might be the reason for the positive tendency in horizontal advection processes. Note that the magnitude of horizontal salt flux shows a reduction when ML salinity shows an enhancement during the daytime (Figs. 9a and S4). The sub-daily evolution of horizontal salt flux estimated from uCTD and SMAP shows good temporal correspondence with the estimation from TSG, though the amplitude of the former two estimations is slightly smaller than the latter (Fig. S4). However, between 1000 and 1400 IST, when the ML salinity shows an increasing trend, the horizontal advection estimated from uCTD and SMAP SSS shows near-zero value in contrast to a mild positive tendency as apparent in TSG-based estimate (Fig. S4). Both surface freshwater flux and horizontal advection show a positive tendency, though these two terms together cannot explain (0.012 psu day^−1^) the large amplitude of ML salinification events (0.04 psu day^−1^) during the daytime (Fig. 9a).

Vertical salinity profiles show a decreasing trend with depth, suggesting that the vertical processes can freshen the ML (~ − 0.03 psu day^−1^) through the entrainment of relatively low saline sub-surface water to the ML during the deepening phase of ML during the night (Figs. 4b and 9a). The vertical processes term shows a positive tendency of magnitude (0.05 psu day^-1^) during the daytime due to the incorporation of detrainment term in the ML salinity, and its magnitude is much higher than horizontal advection and surface freshwater flux (Figs. 4b and 9a). These characteristics suggest that, during the daytime, the re-stratification of ML due to intense solar heating suppresses the entrainment of sub-surface low saline water to ML.

Our ML salinity budget shows that the near-surface salinification during the afternoon is primarily due to a reduction in the entrainment of sub-surface low saline water to ML owing to re-stratification of ML along with the small positive value of evaporation and horizontal advection and a significant positive contribution from detrainment processes.

## Discussion and conclusion

The general notion is that the near-surface ocean surface boundary layer region is not conducive to forming double diffusion processes due to the persistent occurrence of strong turbulent mixing due to wind and buoyancy forces. However, the analysis of a 16-day time-series of the vertical profile of microstructure measurements in the NEAS during spring (7–22 May 2019) shows the existence of salt finger form of double diffusion in the DT region in the afternoon (1000–1900 IST).

Our microstructure analysis shows that diapycnal diffusivity (*K*_*ρ*_) of magnitude ~ 3.4 × 10^−5^ m^2^ s^−1^ in the DT region is primarily due to suppression of the downward penetration of turbulence associated with enhancement of daytime stratification in the near-surface layer by solar radiation. In addition, approximately 80% of *Re*_*b*_ values fall below 100 with a median of 33.3. The low values of *K*_*ρ*_ and *Re*_*b*_ in the DT region suggest the existence of weak shear-driven mixing. It is also found that salinity and temperature decrease with depth in the DT region due to enhancing near-surface salinity and temperature compared to sub-surface during the daytime.

Our analysis shows that the co-existence of relatively weak shear-driven mixing and Turner angle values between 50° and 55° are favorable hydrographic conditions for the formation of salt finger form of double diffusion in the DT region. In addition, the presence of staircase-like structures along with the higher value of *γ*_*χε*_ (> 0.5) compared to *γ*_*Rf*_ (0.2) reinforce our arguments on the existence of salt finger in the DT region of NEAS. It is also found that the mean layer height (0.7 m) is approximately a factor of four higher than the Ozmidov turbulence length scale (0.15 m), suggesting that the staircase-like structure detected in the DT region is associated with salt fingering.

Our ML salinity budget shows that the near-surface salinification (0.04 psu day^−1^) during the afternoon is primarily due to a reduction in the entrainment of sub-surface low saline water to ML owing to re-stratification of ML along with the small positive value of evaporation (0.009 psu day^−1^) and horizontal advection (0.001 psu day^−1^) and a significant positive contribution from detrainment processes (0.05 psu day^−1^).

Note that the present study uses relatively coarse-resolution microstructure data primarily due to faster profiling speed. Hence, high-resolution microstructure measurements, particularly from a platform with relatively slow profiling speed and direct numerical simulations experiments, can provide more insight into the evolution of salt fingering in the surface boundary layer region. These topics are subject to future study.

To the best of our knowledge, the present study demonstrates the first microstructure-based evidence on salt fingers' existence in the DT region. Most importantly, the present study also highlights the possibility of the occurrence of double-diffusive instability immediately after the suppression of strong mechanical-driven turbulence in the surface boundary layer region if hydrographic conditions are favorable. The formation of the salt finger in the surface boundary layer region reported here is a potentially significant result and applicable to wide parts of the tropical ocean where near-surface salinity increases compared to sub-surface in conjunction with the formation of strong DT.

## Methods

### Data

#### Field campaign and microstructure measurements

A sixteen-day time-series measurement of turbulence scale velocity shear, temperature microstructure, and an accurate standard conductivity-temperature (CT) and pressure profiling were carried out in the core of Arabian Sea high salinity water (~ 36.9 psu) at 18.4° N and 67.4° E during 7–22 May 2019 using the research vessel Sagar Kanya (SK-358) (Fig. 1a). The microstructure measurements were made using a free-fall profiler of Rockland Scientific Vertical Microstructure Profiler-250 (VMP-250).

The VMP-250 was equipped with two airfoilshear probes to measure microstructure shear, one fast response thermistor (FP07) to measure microstructure temperature and standard JFE Advantech conductivity-temperature sensors. The sampling rate of microstructure sensors is 512 Hz, and JFE Advantech conductivity-temperature sensor is 64 Hz. The VMP-250 is a free-fall profiler, and an approximate descent rate during the cruise was around 0.8 ms^−1^ and recorded data internally. Our objective is to document salt fingers' existence in the DT, which is typically located between 8 and 18 m of the water column during the daytime. Hence though measurements are available up to 400 m, we restricted ourselves the analysis only to the upper 20 m of the water column. The VMP measurements were carried out from the ship's starboard side when the ship was freely drifting toward the port side. Hence, the possibility of propeller-induced turbulence does not impact our analysis. However, VMP data above 7 m have not been considered for the analysis to avoid the plausible contamination of data associated with the generation of artificial turbulence due to ship wake. While estimating salinity, a thermal lag correction of 0.018 s was applied to the conductivity signal using a single-pole low-pass filter with a cutoff frequency of 0.82 Hz to account for the difference in sensor response time between the conductivity and temperature sensors.

The VMP observations were carried out every 3-h interval, and each cast consisted of 4–5 profiles of VMP measurements (Fig. 1c,d). A total of 623 VMP profiles were collected during this period. Note that one of the primary objectives of this measurement was to examine the existence of double diffusion in the DT region during the daytime between 1000 and 1900 IST. Hence, to resolve the temporal evolution of hydrographic conditions in the DT layers, the VMP measurements were not interrupted by uCTD transects between 1000 and 1900 IST (Fig. 1d; shaded in grey).

#### Satellite data

The eight-day running average of daily sea surface salinity (SSS) data from Soil Moisture Active Passive (SMAP) with 25 km spatial resolution^29^ and Optimally Interpolated (OI) microwave and infrared (MW_IR) daily gridded SST products with 9 km spatial resolution^30^ are used to understand the large scale synoptic condition persist during the study period in the NEAS.

### Salt finger diagnostics

#### Turner angle (Tu)

The Turner angle $\left( {T_{u} \, = \,atan\;(\alpha \Delta T\, + \,\beta \Delta S)/(\alpha \Delta T - \beta \Delta S))} \right)$, an alternate form of density ratio* (R*_*ρ*_ = *α*Δ*T*/*β*Δ*S*) is used to document the existence and relative strength of double diffusion in the NEAS^10,31,32^; where *α* and* β* are thermal expansion, and saline contraction coefficients and Δ*T* and Δ*S* represent the vertical gradients of temperature and salinity. In this study, *Tu* values are estimated based on temperature and salinity measurements with 1 m vertical resolution. The *Tu* values between 45° and 90° (*R*_*ρ*_ > 1) indicate the salt finger form of double diffusion in the water column, whereas the values between − 45° and − 90° (0 < *R*_*ρ*_ < 1) indicate the existence of diffusive convection form of double diffusion^10^.

#### The turbulent kinetic energy dissipation rate (ε)

*ε* was estimated from the shear microstructure data using the expression:

1 $$\varepsilon = {7}.{5}\nu \left\langle {\left( {\partial u^{\prime } /\partial z} \right)^{2} } \right\rangle$$

where ν is the viscosity of seawater, $\left\langle {\left( {\partial u^{\prime } /\partial z} \right)^{2} } \right\rangle$ is the variance of small-scale velocity gradient; <  > represents an average over a finite depth interval. The noise associated with instrument vibrations in the shear data is removed using the Goodman coherent noise removal algorithm^33^. Following Macoun and Lueck^34^ the wavenumber response of the shear probe is corrected by multiplying the measured spectrum by the factor *K*(*k*), and it is estimated using the expression (2)

2 $$K\left( k \right) = 1 + \left( \frac{k}{48} \right)^{2}$$

where k is the wavenumber expressed in units of cpm.

The *ε* is estimated by combining the spectral integration and curve fitting using the dimensional Nasmyth spectrum of the shear power spectrum^35^. The *ε* was estimated from each probe in 4 s bins with a 50% overlap so that the typical vertical resolution of the estimation remained as ~ 1.5 m. Each dissipation length was divided into FFT lengths of 1.3 s with a 50% overlap to improve the statistical reliability of the estimation**.**

#### Estimation of diapycnal diffusivity (K_ρ_)

*K*_*ρ*_ associated with the shear-driven turbulence was estimated using the expression:

3 $$K_{\rho } = \gamma_{Rf} \frac{\varepsilon }{{N^{2} }}$$

where *γ*_*Rf*_ is the mixing coefficient^13,14^, and following Osborn^13^ turbulence model, a constant value of 0.2 is used for the *γ*_*Rf*_. *N*^*2*^ is Brunt–Väisälä frequency, and it is estimated using the expression

4 $$N^{2} = \frac{g}{{\rho_{\theta } }}\frac{{\partial \rho_{\theta } }}{\partial z}$$

where *g* is the acceleration due to gravity (9.8 m s^−2^) and the ${\rho }_{\theta }$ is the potential density of the water column.

#### Buoyancy Reynolds number (***Re***_***b***_)

To characterize the turbulence nature of the water column, Buoyancy Reynolds number (*Re*_*b*_) was estimated using the expression

5 $$Re_{b} = \frac{\varepsilon }{{\nu N^{2} }}$$

where υ is kinematic viscosity (m^2^ s^−1^). As suggested by Bouffard and Boegman^36^, *Re*_*b*_ values below 20 represent the presence of very weak buoyancy-controlled turbulence. The *Re*_*b*_ values between 20 and 100 indicate the transition between weak and energetic turbulent regimes, and values above 100 indicate the occurrence of energetic turbulence in the water column.

#### Ozmidov scale (L_o_)

 The largest length scale of turbulence associated with shear instability in a stably stratified water column is examined using Ozmidov length scale (*L*_*o*_)^37,38^, and it is estimated using the expression

6 $$L_{o} = \left( {\frac{\varepsilon }{{N^{3} }}} \right)^{0.5}$$

#### The thermal variance dissipation rate (*χ)*

*χ* was estimated using the expression

7 $$\chi = 6\kappa_{T} \left\langle {\left( {\partial T^{\prime } /\partial z} \right)^{2} } \right\rangle$$

$\left\langle {\left( {\partial T^{\prime } /\partial z} \right)^{2} } \right\rangle$ is the variance of a small-scale temperature gradient; <  > represents an average over a finite depth interval^39^. We used two distinct methods to calculate χ depending on the value of ε derived from the VMP shear probes^39^. In the low-energy environments (ε ≤ 5 × 10^−8^ W kg^−1^), *χ* is estimated through the integration of temperature gradient spectra in the viscous-diffusive sub-range and in the energetic environments (ε > 5 × 10^−8^ W kg^−1^), *χ* is estimated by fitting Kraichnan’s model spectra in the inertial-convective subrange of the temperature gradient spectra^39^. The estimation obtained through spectral integration and curve-fitting approaches is combined to get the complete profile of *χ*. Similar to the estimation of *ε,* the typical vertical resolution of the estimation *χ* is ~ 1.5 m.

Before fitting to the Kraichnan spectrum, the temperature gradient spectra are corrected using a double-pole frequency response function *F*^2^(*f*) of thermistor^40^

8 $$F^{2} \left( f \right) = \left[ {1 + \left( {2\pi \tau f} \right)^{2} } \right]^{ - 2}$$

where τ is the double pole response time for the FP07 temperature sensor, and it is dependent on profiler speed (*w*), and *τ*_*0*_, a constant primarily associated with glass coating of individual FP07 sensors^41,42^ and τ is estimated using the expression

9 $$\tau = \tau_{0} {\text{w}}^{{ - 0.{5}}} {\text{u}}_{0}^{{0.{5}}}$$

In expression (9) a constant value of 4.1 × 10^−3^ s is prescribed for *τ*_*0*_ and 1 ms^−1^ for *u*_*0*_^39,42^.

#### Mixing coefficient (γ_χε_)

The concurrent availability of *χ* and *ε* estimation from the microstructure measurements were used to estimate *γ*_*χε*_^14,43^, and it can be expressed as

10 $$\gamma_{\chi \varepsilon } = \chi N^{2} /2\varepsilon (\partial T/\partial z)^{2}$$

The uncertainty associated with the estimation of *χ* and* ε* as discussed in the subsequent section may influence the estimation of *γ*_*χε*_. Nevertheless, to corroborate the reliability of *γ*_*χε*_ values in the salt finger regime in the DT region, an alternate form of the mixing coefficient (defined as *γ*_*Rρ*_) is estimated using the following expression^7,44^

11 $$\gamma_{{R_{\rho } }} = \left( {\frac{{R_{\rho } - 1}}{{R_{\rho } }}} \right)\left( {\frac{r}{r - 1}} \right)$$

*r* is estimated using *R*_*ρ*_ in the salt finger regime as presented below^45^

12 $$r = 0.35\exp \left( {1.05\exp \left( { - 2.16\left( {R_{\rho } - 1} \right)} \right)} \right)$$

#### Uncertainty in estimation of *ε* and* χ*

Prescribing an exact value of *τ*_*0*_ in the frequency response function of the thermistor is still a challenging problem^41,46^. Hence, χ is generally reported based on the measurements from the profiler with slower descending speed and/or regions of low dissipation rates^41^. We evaluated the uncertainty associated with the estimation of *χ* in the DT region by assigning different values (3 × 10^−3^ s and 5 × 10^−3^ s) compared to our default choice of 4.1 × 10^−3^ s. It is found that the estimation of *χ* based on the values of 3 × 10^−3^ s and 5 × 10^−3^ s for *τ*_*0*_ differed by a factor of 1.2 with respect to our default choice.

In addition, the existence of a sharp gradient in temperature and shear associated with the interface can lead to the overestimation of *ε* and *χ* in salt finger staircase-like structure. The previous studies demonstrated that the estimation of *χ* in the salt finger interfaces was overestimated by a factor of 4–20 compared to its values in the layers alone^19,44,47^. However, such an overestimation is relatively small in the estimation of *ε* (approximately a factor of 1.5) compared to *χ*^19,44,47^^.^ One plausible way to remove this error from the microstructure data is to estimate *ε* and *χ* in the layer region alone^47^. However, low-vertical resolution microstructure data used in this study, primarily due to faster profiler speed, does not allow us to estimate *ε* and *χ* only in the layers in the DT region. Previous studies have also shown that the estimation of *ε* and *χ* in the entire staircase (layer and interface) is dominated by values in the layers rather than in the interfaces^44^. Such as, overestimation is approximately a factor of 1.5 (4) for the estimation of *ε* (*χ*) in the entire staircase compared to the layer. Hence, the possible errors associated with the estimation of *ε* and *χ* due to these factors are ignored in the present study. It is found that the staircase structure in the DT region reported in the present study is not well-defined, and the interfaces have similar heights as the staircase layers. The impact of these characteristics on the uncertainty in dissipation parameter estimates compared to the sharp interface in the well-defined staircase is not yet reported, and such details are beyond the scope of the present work.

It is also observed that* ε* and χ change rapidly within 3 m below the ML. Note that ε and χ were estimated from ~ 3 m data segment with a 50% overlap. Hence, the estimated values of ε and χ immediately below the ML might have been influenced by the segment of data from the ML. Note that 65% of values of *ε* in the DT region between 1000 and 1900 IST is below 5 × 10^–8^ W kg^−1^. While it is increased to 85% after discarding the first 2 m data points immediately below the ML in the DT region. These characteristics also suggest that such an approach makes sure our estimation of *χ* in the DT region is primarily through the integration method. Hence, while reporting the value of *K*_*ρ*_ and *γ*_*χε*_ we discarded data points in the first 2 m immediately below the ML. The mean (*µ*) and standard deviation (*σ*) of *K*_*ρ*_ is estimated by maximum likelihood estimates fitting the lognormal distribution to the data. The *µ* and *σ* of the logarithm of data is then converted to the mean and standard deviation of original data using the expression^48,49^,

13 $$Mean \left( m \right) = e^{{\mu + \frac{1}{2}\sigma^{2} }}$$

14 $$Standard deviation = \sqrt {m^{2} \left( {e^{{\sigma^{2} }} - 1} \right)}$$

#### Staircase structure detection algorithm

The staircase structures in temperature profiles were identified using the algorithm proposed in the previous studies^2,4^. For this purpose, the individual temperature profiles from VMP CTD were gridded into 0.1 m vertical resolution, and the background temperature gradient was estimated from this data by taking a 2.5 m running mean^4^. A layer (*H*) is defined as the portion of the temperature profile where at least two temperature gradient values are smaller than the background gradient, and the smallest value of these gradients should be less than the background gradient by at least 0.02 °C m^−1^. We consider a staircase structure only if the interface occurs immediately after a layer. The portion of the temperature profile where at least one temperature gradient value greater than the background gradient by 0.02 °C m^−1^ is qualified as an interface. The distance between the base of the layer immediately above the interface and the base of the interface is defined as interface height (*h*). The temperature change across the interface is represented as Δ*T*_*h*_. We used only the temperature profile to identify the staircase structure in the DT region. The concurrent staircase-like structure can occur in temperature and salinity profiles in a well-defined double diffusion regime. In the DT region, the staircase-like structure is not well-defined. As a result, it is possible that the staircase in the salinity profile may not always coincide with the steps in the temperature. However, as depicted in Fig. 3d, the coherence in the staircase-like structure is evident in the temperature and salinity profile. It is unlikely that such a situation may always exist.

### Mixed layer depth

The ML depth (MLD) is defined as the depth where the temperature is 0.1 °C greater than the surface value, the selection of this criteria is to resolve the diurnal variability of MLD and demarcate the DT region during the study period (supporting information; Figs. S6 and S7).

### ML salinity budget

#### ML salinity budget equation

We considered a simplified version of the ML salinity budget^50^

15 $$\frac{\partial S}{{\partial t}} = \frac{{\left( {E - P} \right)S }}{MLD} - \left[ {u\frac{\partial S}{{\partial x}} + v\frac{\partial S}{{\partial y}}} \right] - \left[ {W_{MLD} + \frac{\partial MLD}{{\partial t}}} \right]\frac{{\left( {S - S_{MLD} } \right)}}{MLD} \; + \;{\text{Residual}}$$

In Eq. (15), terms from left to right represent the rate of change of ML averaged salinity tendency, local freshwater flux due to the difference between evaporation (E) and precipitation (P), horizontal advection, vertical processes due to the vertical advection below the base of ML (*W*_*MLD*_) and rate of change of MLD ($\frac{\partial MLD}{\partial t}$), and residual.

The motivation of the near-surface salinity budget in the present study is to investigate the plausible causative mechanism responsible for the enhancement of salinity in the region consisting of ML and DT rather than just for the ML. For that purpose, a depth was estimated where the temperature is 0.3 °C lower than the surface value, and this criterion reasonably captured the base of DT (Fig. 5a,b; supporting information; Figs. S6 and S7). We defined this near-surface region salinity budget as “ML salinity budget” throughout the manuscript for brevity. Note that 0.3 °C temperature criterion is used only for the near-surface salinity budget in the present study; otherwise, our discussion is based on ML criterion of 0.1 °C through out the manuscript.

#### Net heat flux and local freshwater flux

The evaporation (E) from the sea surface is estimated using the expression.

16 $$E = LHF\rho_{w}^{ - 1} L_{e}^{ - 1}$$

where *LHF* is latent heat flux, *ρ*_*w*_ is the density of seawater, and *L*_*e*_ is the latent heat of vaporization. *LHF* is estimated using Coupled Ocean–Atmosphere Response Experiment (COARE 3.6) bulk flux algorithm using *SST*, relative humidity, air temperature, wind speed, downwelling longwave radiation (*DLW*)*,* and downwelling shortwave radiation (*DSW)*^51,52^. The diurnal warm layer and cool skin correction were prescribed for *SST* measurements while estimating *LHF*^51^. *DLW*, air temperature, and humidity data from the ship-mounted automatic weather station fixed at 14 m above the sea surface and SST (0.5 m), wind speed (at the height of 3 m), and *DSW* (at the height of 3 m) data from the Ocean Moored buoy Network for the Northern Indian Ocean (OMNI) mooring^53^ located approximately 2 km away from the VMP time-series station (~ 18.4° N and 67.4° E; AD06) with the one-hour temporal resolution are used to estimate *LHF*. The dome heating effect was removed from the DLW data using the expression *DLW*-DSW*0.036^54^. The precipitation data from the AWS system is used to facilitate the analysis; however, it is worth pointing out that no precipitation events were identified during the study period. The *L*_*e*_ is estimated from mooring SST using the expression

17 $$L_{{\text{e}}} = \left( {{2}.{5}0{1} - 0.00{237}\; \times \;SST} \right)\; \times \;{1}0^{{6}}$$

An albedo correction of 5.5% is applied to *DSW* (*DSW* × 0.945) to estimate *NSW*. Net longwave radiation (*NLW*) is estimated as a difference between the upward and downward components of longwave radiation. The upward component of longwave radiation is estimated using the expression 0.97*σSST*^4^, where the constant 0.97 is the broadband emissivity of seawater and* σ* is the Stefan-Boltzmann constant (5.67 × 10^−8^ W m^−2^ K^−4^).

The penetrating component of shortwave radiation (*Q*_*Pen*_) below the ML is estimated by using the expression^55,56^,

18 $$Q_{Pen} = 0.47NSW\left[ {V_{1} e^{{ - MLD/\zeta_{1} }} + V_{2} e^{{ - MLD/\zeta_{2} }} } \right]$$

where* ζ*_*1*_ (~ 19 m)*,* and *ζ*_*2*_ (~ 5 m) is the attenuation depths of long visible and short visible and ultraviolet wavelengths, respectively. The fraction of 0.47 indicates that 53% of the infrared component (> 750 nm) of the *NSW* is absorbed within 2 m depth, and the remaining 47% penetrates to the deeper water column. *V*_*1*_ (~ 38%) and *V*_*2*_ (~ 62%) represent the fraction of the long visible and short visible and ultraviolet wavelengths after removing the infrared component from the *NSW* that is absorbed within 1–2 m of the water column*.* Chlorophyll*-a* (mg m^−3^) data obtained from a WETLAB sensor-operated during every day at 1700 IST at the time-series station is used to estimate the values of *V*_*1*_, *V*_*2*_, *ζ*_*1*_, and *ζ*_*2*_*.*

The net surface heat flux (*NHF)* is estimated as the sum of *LHF*, *SHF*, *NSW*-*Q*_*pen*_*,* and *NLW*. We followed the convention, such as the positive values of heat flux indicating the ocean’s heat gain.

#### Vertical processes

The vertical process term in Eq. (15) consists of vertical advection, entrainment during the deepening phase of ML, and detrainment during the shallowing phase of ML. the entrainment/detrainment velocity at the base of ML is estimated as the rate of change of MLD ($\frac{\partial MLD}{\partial t}$). Theoretically, the properties in the ML should be perfectly uniform. In this case, the shallowing of ML does not impact ML salinity since re-stratification processes leave the water at the lower portion of ML, which has the same magnitude as in ML. Hence, in the ideal ML case, the detrainment processes can be ignored in ML salinity budget analysis^57,58^. However, a weak gradient in parameters must have existed in the ML when the ML base is defined through a criterion of small increment in stratification in a depth range. In this case, the water that leaves the ML during the stratification phase of ML has a relatively different salinity than ML averaged salinity. Hence, following Kim et al.^57^ and Cronin et al.^58^, the detrainment term is retained along with the entrainment term for the better accuracy of the ML budget in the study. The vertical advection below the base of ML (*W*_*MLD*_) is estimated from the rate of change of 27 °C isotherms in the thermocline^59,60^. *S*_*MLD*_ is the salinity of water entrained into the ML, taken to be salinity at 1 m below MLD estimated based on 0.3 °C temperature criterion^60^.

#### Horizontal advection

Current velocity measurements from a Doppler Volume Sampler (DVS) equipped at a depth of 1.5 m in AD06 mooring is used to estimate horizontal advection.

The horizontal gradient of near-surface salinity ($\frac{\partial S}{\partial x},$ $\frac{\partial S}{\partial y}$) was estimated from the meridional and zonal underway CTD (uCTD) and thermosalinograph (TSG) sections traversing through the VMP time series locations (Fig. 1c,d). Two zonal (east-centre and west-centre) and meridional (north-centre and south-centre) transects were carried out every day during the 16-day time series observation (Fig. 1d). The west-centre (north-centre) and east-centre (south-centre) transects were separated by approximately 3 h (Fig. 1c, d). The horizontal distance of 10 km from the endpoint of the zonal and meridional uCTD/TSG transect to the VMP time-series station (e.g., from west to centre) was covered within 1 h (Fig. 1c,d). The uCTD was operated in a free-spool mode from the moving vessel (~ 6 kt), and the typical fall speed of the probe was around ~ 3 ms^−1^. The uCTD samples temperature, conductivity, and pressure at 16 Hz. The typical spatial resolution of salinity measurements using uCTD along the transect is roughly 1 km. The spatial resolution of TSG measurement is around 1 km (~ 5 min).

The spatial gradient of salinity along each transect (e.g., east-centre) is estimated from the slope of a linear least-square fit of uCTD and TSG salinity data at 6 m depth (Fig. S8)^61^. The spatial gradient of salinity estimated from these two instruments shows reasonable good agreement (Fig. S8). The mean value of the salinity gradient estimated from the east-centre and west-centre transect in a day is used as a daily zonal salinity gradient ($\frac{\partial S}{\partial x}$). Similarly, gradient values of north-centre and south-centre transects are averaged to obtain the daily value of the meridional salinity gradient ($\frac{\partial S}{\partial y}$).

In addition, the eight-day composite of daily SMAP SSS averaged over four-grid points (~ 50 km) on either side of the VMP time-series station was also used to estimate the horizontal gradient of SSS.

The daily value of $\frac{\partial S}{\partial x}\mathrm{ and}$ $\frac{\partial S}{\partial y}$ was interpolated to the temporal resolution of VMP observation to facilitate the analysis. In the result and discussion section, our analysis was restricted to the horizontal salt flux estimation based on the spatial salinity gradient estimated from TSG. The horizontal salt flux estimated using SMAP and uCTD lateral salinity gradient is used to evaluate the accuracy of the estimation based on TSG.

Note that no *in-situ* horizontal salinity gradient estimation is available between 1000 and 2200 IST; hence, its impact on the calculation of horizontal advection term during the daytime should be considered with caution (Fig. 1d)**.**

#### Residual

The residual term includes the errors associated with horizontal currents and local surface freshwater fluxes, errors in parameterizing vertical processes, computational errors associated with finite differencing, sampling errors, and neglected physical processes such as horizontal and vertical diffusivity.

#### Monin–Obukhov length

The ratio between MLD and Monin–Obukhov length ($${L}_{MO})$$ ($$\frac{MLD}{{L}_{MO}}$$) is used to examine whether the turbulent mixing over the MLD is driven by wind stress ($$\tau )$$ or buoyancy flux ($${B}_{f}$$)^17,62–64^.

${L}_{MO}$ is estimated using the expression

19 $$L_{MO} = - \frac{{U_{*}^{3} }}{{\kappa B_{f} }}$$

where $\kappa$ is Von Karman’s constant ~ 0.4, ${U}_{*}$ is frictional velocity, and it is estimated from surface wind stress ($\tau$) and density of the seawater (${\rho }_{w}$) using the expression ${\tau }^{1.5}{\rho }_{w}^{-1.5}$. The surface buoyancy flux ($B_{f}$) is estimated using the expression

20 $$B_{f} = g\left( {\frac{ - \alpha NHF}{{\rho_{{0C_{p} }} }} + \beta SSS\left( {E - P} \right)} \right)$$

${B}_{f}$ is defined as negative when the ocean receives positive heat flux and/or excess precipitation over evaporation. Hence, during the convective phase, ${L}_{MO}$ is negative, and values of ($\frac{MLD}{{L}_{MO}}$) << − 1. During the re-stratification phase owing to negative buoyancy flux, the ${B}_{f}$ suppress turbulence below the ML and $\frac{MLD}{{L}_{MO}}$ ≤ 1. However, studies have suggested that due to unaccounted turbulence sources in Monin–Obukhov similarity theory, such as surface gravity waves^65,66^ and formation of the diurnal shear layer during daytime due to trapping of momentum in the near-surface layer during low wind speed and intense solar heating conditions^22,67^ the values of the ratio $\frac{MLD}{{L}_{MO}}$ can be much higher than one during the re-stratification regime^64^.

## Supplementary Information

Supplementary Information.

## Data Availability

All data, code, and materials for processing microstructure data and the analyses are available from girish@incois.gov.in. Data sources for the remaining data are available in the main text or supplementary materials.

## References

[1] Laurent LS, Schmitt RW (1999). The contribution of salt fingers to vertical mixing in the North Atlantic Tracer Release Experiment. J. Phys. Oceanogr..

[2] Sirevaag A, Fer I (2012). Vertical heat transfer in the Arctic Ocean: The role of double-diffusive mixing. J. Geophys. Res. Oceans.

[3] Schmitt RW, Ledwell JR, Montgomery ET, Polzin KL, Toole JM (2005). Enhanced diapycnal mixing by salt fingers in the thermocline of the tropical atlantic. Science.

[4] Walesby K, Vialard J, Minnett PJ, Callaghan AH, Ward B (2015). Observations indicative of rain-induced double diffusion in the ocean surface boundary layer. Geophys. Res. Lett..

[5] Nagai T, Inoue R, Tandon A, Yamazaki H (2015). Evidence of enhanced double-diffusive convection below the main stream of the Kuroshio Extension. J. Geophys. Res. Oceans.

[6] Bebieva Y, Timmermans ML (2016). An examination of double-diffusive processes in a mesoscale eddy in the Arctic Ocean. J. Geophys. Res. Oceans.

[7] Hamilton JM, Lewis MR, Ruddick BR (1989). Vertical fluxes of nitrate associated with salt fingers in the world’s oceans. J. Geophys. Res..

[8] Vladoiu A, Bouruet-Aubertot P, Cuypers Y, Ferron B, Schroeder K, Borghini M, Leizour S, Ismail SB (2019). Mixing efficiency from microstructure measurements in the Sicily Channel. Ocean Dyn..

[9] Nakano H, Yoshida J (2019). A note on estimating eddy diffusivity for oceanic double-diffusive convection. J. Oceanogr..

[10] You Y (2002). A global ocean climatological atlas of the Turner angle: Implications for double-diffusion and water-mass structure. Deep Res. Part I Oceanogr. Res. Pap..

[11] Van der Boog CG, Dijkstra HA, Pietrzak JD, Katsman CA (2021). Double-diffusive mixing makes a small contribution to the global ocean circulation. Commun. Earth Environ..

[12] Fernández-Castro B, Mouriño-Carballido B, Marañón E, Chouciño P, Gago J, Ramírez T, Vidal M, Bode A, Blasco D, Royer SJ, Estrada M, Simó R (2015). Importance of salt fingering for new nitrogen supply in the oligotrophic ocean. Nat. Commun..

[13] Osborn TR (1980). Estimates of the local rate of vertical diffusion from dissipation measurements. J. Phys. Oceanogr..

[14] Gregg MC, D’Asaro EA, Riley JJ, Kunze E (2018). Mixing efficiency in the ocean. Ann. Rev. Mar. Sci..

[15] Soloviev A, Vershinsky N (1982). The vertical structure of the thin surface layer of the ocean under conditions of low wind speed. Deep Sea Res. Part A Oceanogr. Res. Pap..

[16] Soloviev A, Lukas R (1997). Observation of large diurnal warming events in the near-surface layer of the western equatorial Pacific warm pool. Deep Res. Part I Oceanogr. Res. Pap..

[17] Lombardo CP, Gregg MC (1989). Similarity scaling of viscous and thermal dissipation in a convecting surface boundary layer. J. Geophys. Res. Oceans.

[18] Sutherland G, Marié L, Reverdin G, Christensen KH, Broström G, Ward B (2016). Enhanced turbulence associated with the diurnal jet in the ocean surface boundary layer. J. Phys. Oceanogr..

[19] Gregg MC, Sanford TB (1987). Shear and turbulence in thermohaline staircases. Deep Sea Res. Part A Oceanogr. Res. Pap..

[20] Stellmach S, Traxler A, Garaud P, Brummell N, Radko T (2011). Dynamics of fingering convection. Part 2 the formation of thermohaline staircases. J. Fluid Mech..

[21] Linden PF (1978). The formation of banded salt finger structure. J. Geophys. Res. Oceans.

[22] Price JF, Weller RA, Pinkel R (1986). Diurnal cycling: Observations and models of the upper ocean response to diurnal heating, cooling, and wind mixing. J. Geophys. Res. Ocean..

[23] Bernie DJ, Guilyardi E, Madec G, Slingo JM, Woolnough SJ, Cole J (2008). Impact of resolving the diurnal cycle in an ocean-atmosphere GCM. Part 2: A diurnally coupled CGCM. Clim. Dyn..

[24] Wenegrat JO, McPhaden MJ (2015). Dynamics of the surface layer diurnal cycle in the equatorial Atlantic Ocean (0°, 23°W). J. Geophys. Res. Oceans.

[25] Drushka K, Gille ST, Sprintall J (2014). The diurnal salinity cycle in the tropics. J. Geophys. Res. Oceans.

[26] Cronin MF, McPhaden MJ (1999). Diurnal cycle of rainfall and surface salinity in the western Pacific warm pool. Geophys. Res. Lett..

[27] Joseph J, Girishkumar MS, McPhaden MJ, Rao EPR (2021). Diurnal variability of atmospheric cold pool events and associated air-sea interactions in the Bay of Bengal during the summer monsoon. Clim. Dyn..

[28] Johnson RH, Ciesielski PE (2017). Multiscale variability of the atmospheric boundary layer during DYNAMO. J. Atmos. Sci..

[29] Meissner T, Wentz FJ, Le Vine DM (2018). The salinity retrieval algorithms for the NASA aquarius version 5 and SMAP version 3 releases. Remote Sens..

[30] Gentemann, C. L., Wick, G. A., Cummings, J. & Bayler, E. Multi-sensor improved sea surface temperature (MISST) for GODAE. in *Conference on Satellite Meteorology and Oceanography* 79–81 (2004).

[31] Turner JS (1973). Buoyancy Effects in Fluids.

[32] McDougall TJ (1988). Small-scale turbulence and mixing in the ocean: A glossary. Elsevier Oceanogr. Ser..

[33] Goodman L, Levine ER, Lueck RG (2006). On measuring the terms of the turbulent kinetic energy budget from an AUV. J. Atmos. Ocean. Technol..

[34] Macoun P, Lueck R (2004). Modeling the spatial response of the airfoil shear probe using different sized probes. J. Atmos. Ocean. Technol..

[35] Lueck R (2015). Calculating the rate of dissipation of turbulent kinetic energy. RSI Tech. Note.

[36] Bouffard D, Boegman L (2013). A diapycnal diffusivity model for stratified environmental flows. Dyn. Atmos. Oceans.

[37] Ozmidov RV (1965). On the turbulent exchange in a stably stratified ocean. Izv. Acad. Sci. USSR Atmos. Ocean. Phys..

[38] Dillon TM (1982). Vertical overturns: A comparison of Thorpe and Ozmidov length scales. J. Geophys. Res. Oceans.

[39] Bluteau CE, Lueck RG, Ivey GN, Jones NL, Book JW, Rice AE (2017). Determining mixing rates from concurrent temperature and velocity measurements. J. Atmos. Ocean. Technol..

[40] Gregg MC, Meagher TB (1980). The dynamic response of glass rod thermistors. J. Geophys. Res. Oceans.

[41] Gregg MC (1999). Uncertainties and limitations in measuring ε and χ(T). J. Atmos. Ocean. Technol..

[42] Vachon, P. & Lueck, R. G. A small combined temperature-conductivity probe. in *Proc. of the 1984 STD Conference and Workshop* 126–131 (1984).

[43] Oakey NS, Elliott JA (1982). Dissipation within the surface mixed layer. J. Phys. Oceanogr..

[44] McDougall TJ, Ruddick BR (1992). The use of ocean microstructure to quantify both turbulent mixing and salt-fingering. Deep Sea Res. Part A Oceanogr. Res. Pap..

[45] Kelley D. *Oceanic Thermocline Staircase*. PhD thesis (1986).

[46] Hummels, R. M. *On the Variability of Turbulent Mixing Within the Upper Layers of the Atlantic Cold Tongue Region*. PhD thesis 153 (2012).

[47] Guthrie JD, Fer I, Morison J (2015). Observational validation of the diffusive convection flux laws in the Amundsen Basin, Arctic Ocean. J. Geophys. Res. Oceans.

[48] Fernández-Castro B, Mouriño-Carballido B, Benítez-Barrios VM, Chouciño P, Fraile-Nuez E, Graña R, Piedeleu M, Rodríguez-Santana A (2014). Microstructure turbulence and diffusivity parameterization in the tropical and subtropical Atlantic, Pacific and Indian Oceans during the Malaspina 2010 expedition. Deep Sea Res. Part I Oceanogr. Res. Pap..

[49] Baker MA, Gibson CH (1987). Sampling turbulence in the stratified ocean: Statistical consequences of strong intermittency. J. Phys. Oceanogr.

[50] Rao RR, Sivakumar R (2003). Seasonal variability of sea surface salinity and salt budget of the mixed layer of the north Indian Ocean. J. Geophys. Res. Oceans.

[51] Fairall CW, Bradley EF, Godfrey JS, Wick GA, Edson JB, Young GS (1996). Cool-skin and warm-layer effects on sea surface temperature. J. Geophys. Res. C Oceans.

[52] Edson JB, Jampana V, Weller RA, Bigorre SP, Plueddemann AJ, Fairall CW, Miller SD, Mahrt L, Vickers D, Hersbach H (2013). On the exchange of momentum over the open ocean. J. Phys. Oceanogr..

[53] Venkatesan R, Shamji VR, Latha G, Mathew S, Rao RR, Muthiah A, Atmanand MA (2013). In situ ocean subsurface time-series measurements from OMNI buoy network in the Bay of Bengal. Curr. Sci..

[54] Dickey TD, Manov DV, Weller RA, Siegel DA (1994). Determination of longwave heat flux at the air-sea interface using measurements from buoy platforms. J. Atmos. Ocean. Technol..

[55] Foltz GR, Vialard J, Kumar BP, McPhaden MJ (2010). Seasonal mixed layer heat balance of the southwestern tropical Indian Ocean. J. Clim..

[56] Sweeney C, Gnanadesikan A, Griffies SM, Harrison MJ, Rosati AJ, Samuels BL (2005). Impacts of shortwave penetration depth on large-scale ocean circulation and heat transport. J. Phys. Oceanogr..

[57] Kim SB, Fukumori I, Lee T (2006). The closure of the ocean mixed layer temperature budget using level-coordinate model fields. J. Atmos. Ocean. Technol..

[58] Cronin MF, Pelland NA, Emerson SR, Crawford WR (2015). Estimating diffusivity from the mixed layer heat and salt balances in the North Pacific. J. Geophys. Res. Ocean..

[59] McPhaden MJ (1982). Variability in the central equatorial Indian Ocean. Part I: Ocean dynamics. J. Mar. Res..

[60] Girishkumar MS, Joseph J, Thangaprakash VP, Pottapinjara V, McPhaden MJ (2017). Mixed layer temperature budget for the northward propagating summer monsoon intraseasonal oscillation (MISO) in the central Bay of Bengal. J. Geophys. Res. Ocean..

[61] Wijesekera HW, Rudnick DL, Paulson CA, Pierce SD, Pegau WS, Mickett J, Gregg MC (2005). Upper ocean heat and freshwater budgets in the eastern Pacific warm pool. J. Geophys. Res. C Ocean..

[62] Monin AS, Obukhov AM (1954). Basic laws of turbulent mixing in the surface layer of the atmosphere. Contrib. Geophys. Inst. Acad. Sci. USSR.

[63] Weller RA, Fischer AS, Rudnick DL, Eriksen CC, Dickey TD, Marra J, Fox C, Leben R (2002). Moored observations of upper-ocean response to the monsoons in the Arabian Sea during 1994–1995. Deep Res. Part II Top. Stud. Oceanogr..

[64] Sutherland G, Reverdin G, Marié L, Ward B (2014). Mixed and mixing layer depths in the ocean surface boundary layer under conditions of diurnal stratification. Geophys. Res. Lett..

[65] Craig PD, Banner ML (1994). Modeling wave-enhanced turbulence in the ocean surface layer. J. Phys. Oceanogr..

[66] D’asaro EA (2014). Turbulence in the upper-ocean mixed layer. Ann. Rev. Mar. Sci..

[67] Wenegrat JO, McPhaden MJ (2015). Dynamics of the surface layer diurnal cycle in the equatorial Atlantic Ocean (0°, 23°W). J. Geophys. Res. Ocean..

