# Supplementary material for: Observational evidence of salt finger in the diurnal thermocline
Source: Sci Rep. 2023 Mar 3;13:3627. doi: 10.1038/s41598-023-30564-5 (PMC9984390; doi:10.1038/s41598-023-30564-5)
Supplement: Supplementary file 1 — Supplementary Information. [file 41598_2023_30564_MOESM1_ESM.docx]

**Supplementary Materials for “Observational evidence of salt finger in the diurnal thermocline”**

K. Ashin, M. S. Girishkumar, Eric D'Asaro, Jofia Joseph, V. R. Sherin, N. Sureshkumar, E. Pattabhi Ram Rao

^*^Corresponding author: M. S. Girishkumar, Indian National Centre for Ocean Information Services (INCOIS), Hyderabad—500 055, India. Email: [girish@incois.gov.in](mailto:girish@incois.gov.in)

**This file includes:**

Figures S1 to S9

Text-S1


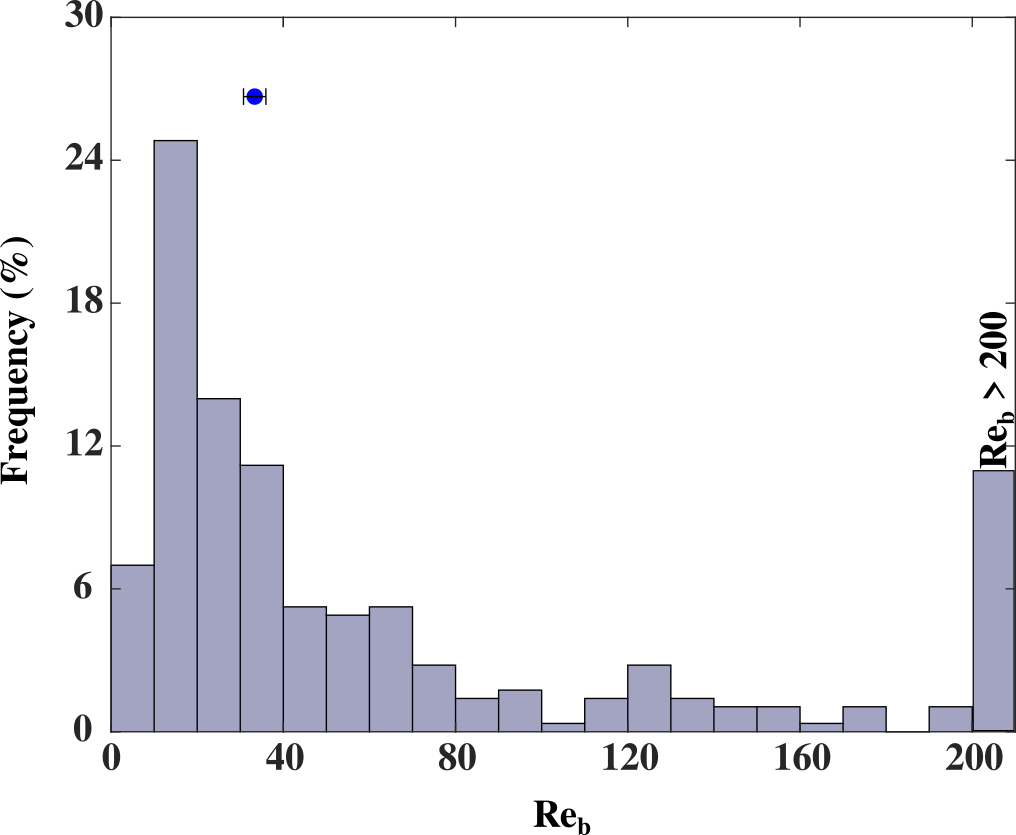


Figure S1. Frequency distribution (%) of *Reb* values between 1000 LST to 1800 LST in the DT region at the VMP time-series station (18.4°N and 67.4°E). The blue circle inside the figure represents the median value of each parameter. The error bar represents one standard error of median. The percentage of data points fall in different *Reb* regimes: *Reb* < 20 - 32% ; 20 < *Reb* <100 - 47%; 100 < *Reb* <200 - 10%; *Reb* > 200 - 11%.


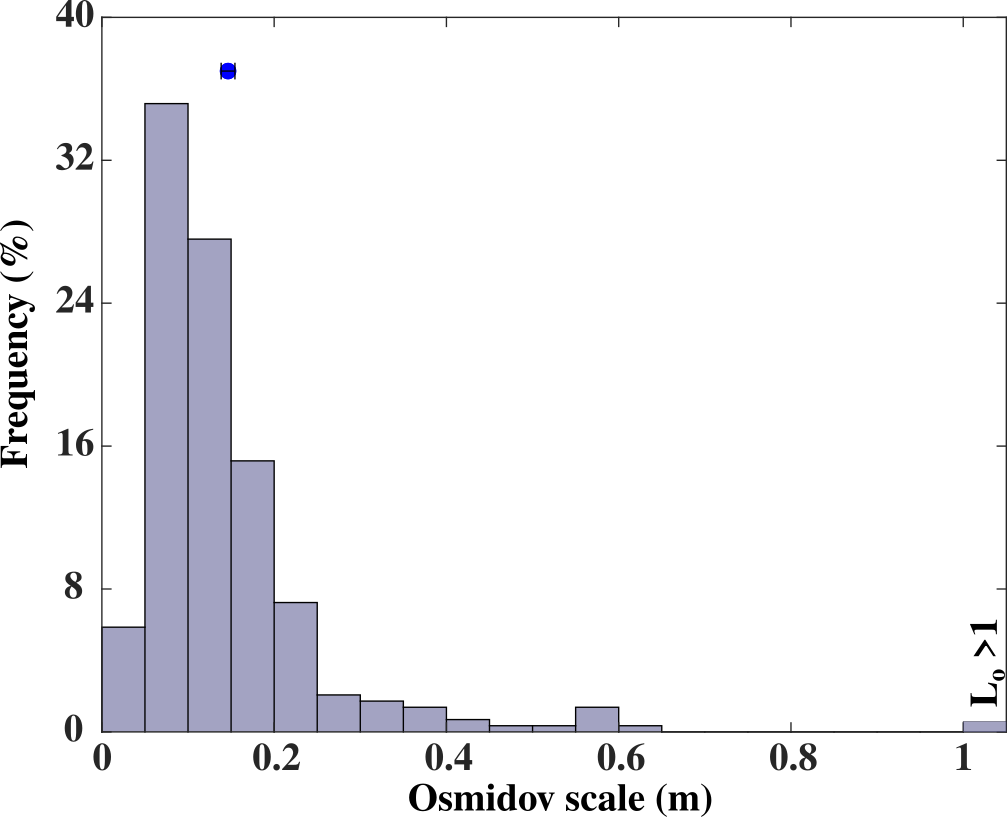


Figure S2. Frequency distribution (%) of Ozmidov scale (*L_o_*_;_ m) values between 1000 LST to 1800 LST in the DT region at the VMP time-series station (18.4°N and 67.4°E).


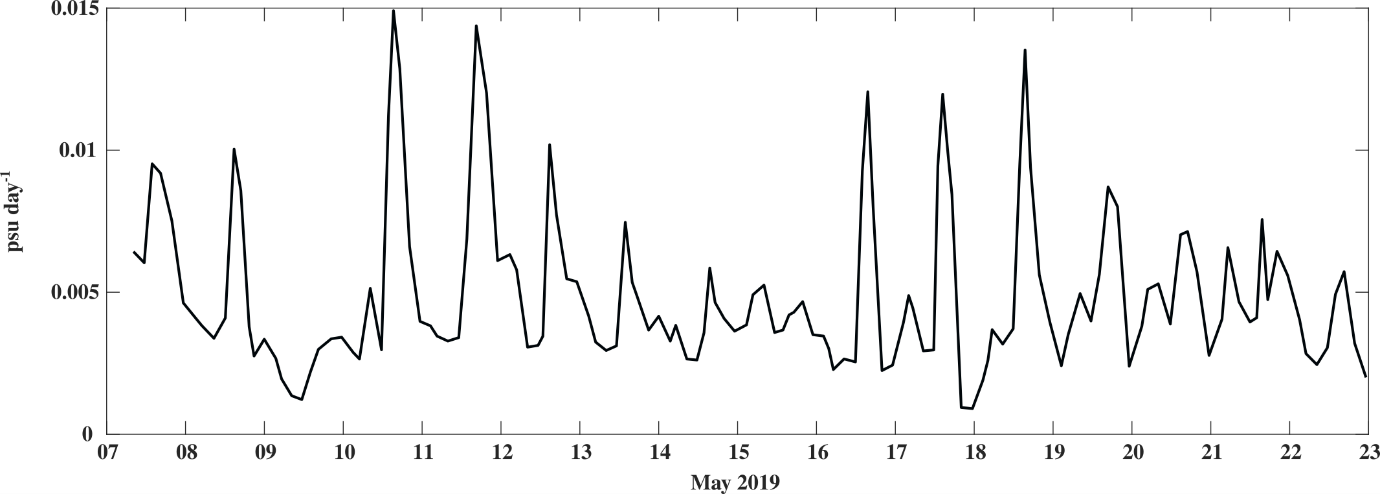


Figure S3. Temporal evolution of freshwater flux due to evaporation (E*S*MLD^-1^; psu day^-1^) during 07-22 May 2019 at VMP time-series station (18.4°N and 67.4°E). The MLD was estimated using the 0.3°C temperature criterion. Well-defined sub-daily variability with an enhancement in evaporation during the daytime is apparent.


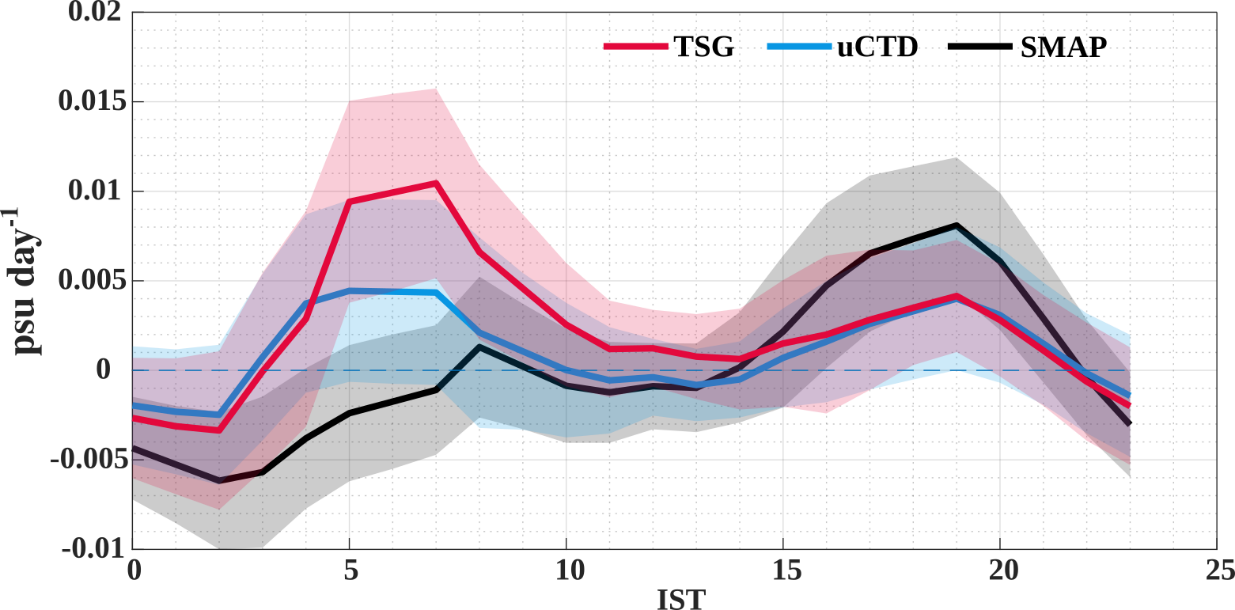


Figure S4. The composite (07-22 May 2019) of sub-daily evolution of horizontal advection term based on the horizontal salinity gradient estimated from TSG (red), uCTD (blue), and gridded SMAP SSS data (black). The shading represents one standard error of the mean, and it is estimated based on the deviations of data from the mean in each 1-hour bin using the bootstrap method. The dashed horizontal line represents the zero value of salinity tendency. Time is in IST hours.


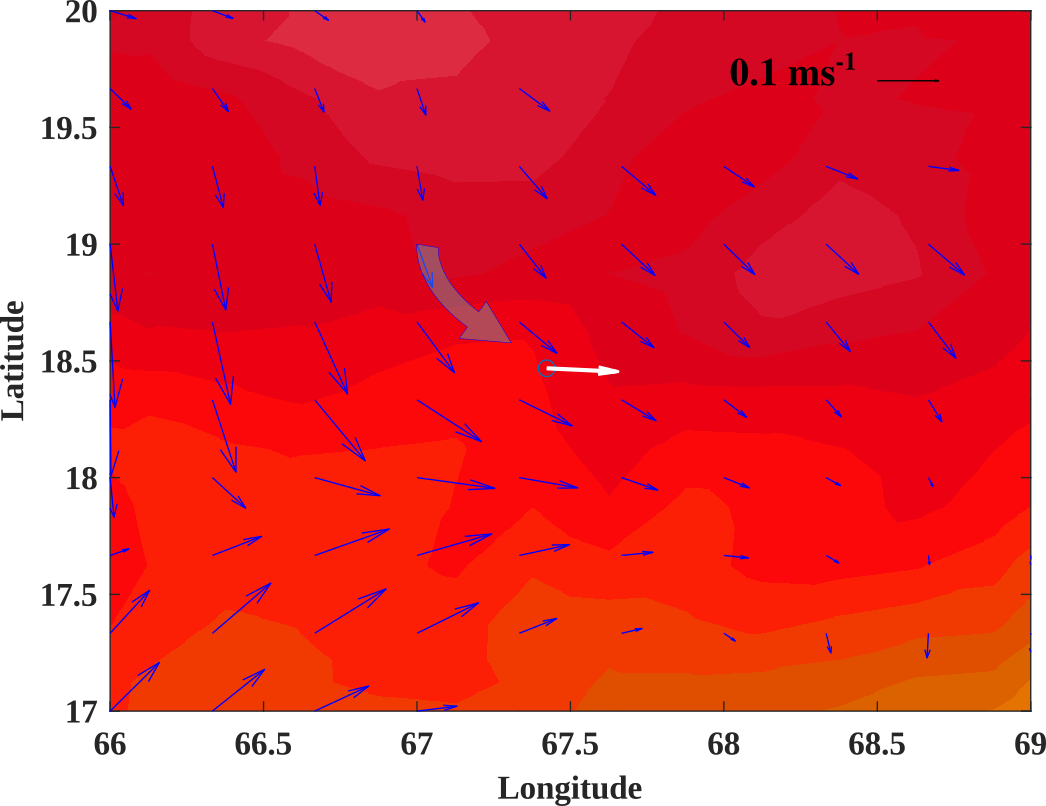


Figure S5. The SMAP SSS (shading) and OSCAR surface current (blue vectors, ms^-1^) averaged during the time series observation period between 7-23 May 2019 in the NEAS. The VMP time-series station is marked as a black open circle. The AD06 surface current (1.2 m) averaged during the time series observation period between 7-23 May 2019 is marked in a white vector. The thick grey arrow represents the schematic of the advection of high saline water from the north to the time-series station through prevailing circulation.


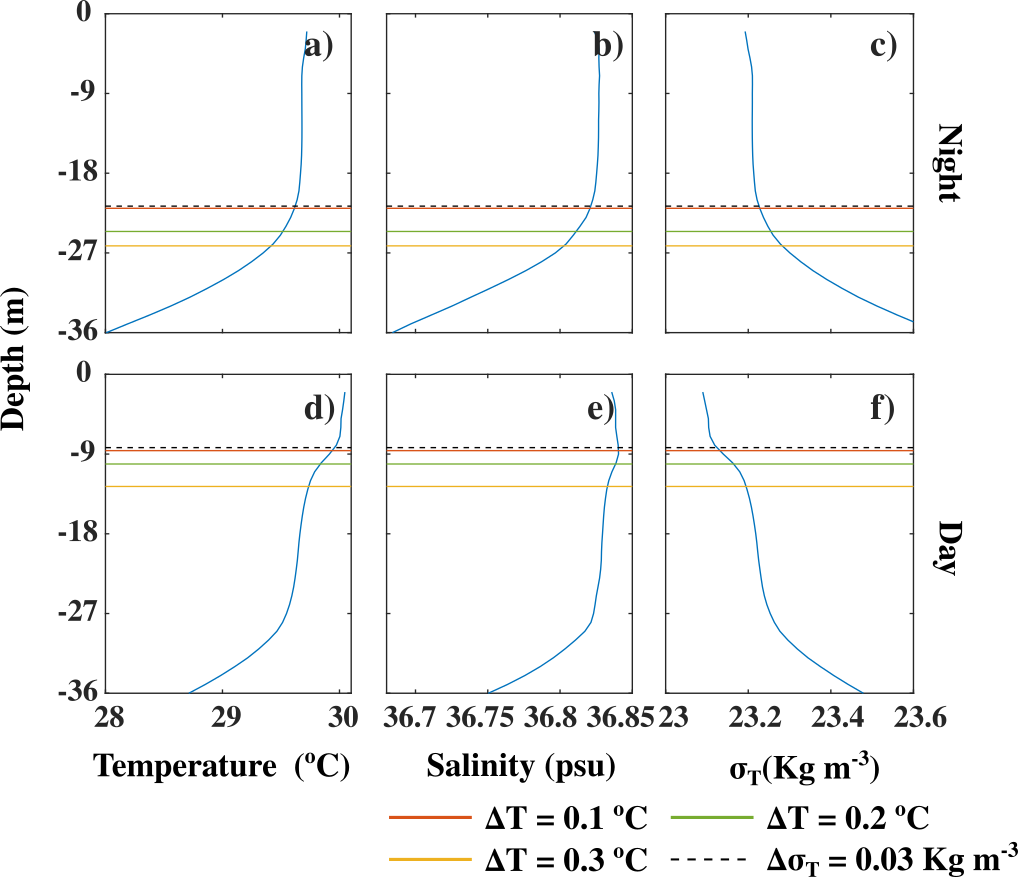


Figure S6. Composite (07-22 May 2019) average of the vertical profile of (a and d) temperature (°C), (b and e) salinity (psu), and (c and f) *σ_t_* (kg m^-3^) during (a, b and c) nighttime (0300 IST) and (d, e and f) afternoon (1500 IST). In the panels, the mixed layer depth estimated using temperature criterion (*∆T*) of the magnitude of 0.1°C (red), 0.2°C (green), 0.3°C (yellow), and density criterion (*∆σ_t_*) of magnitude 0.03 kg m^-3^ (black dashed) are marked as horizontal lines.


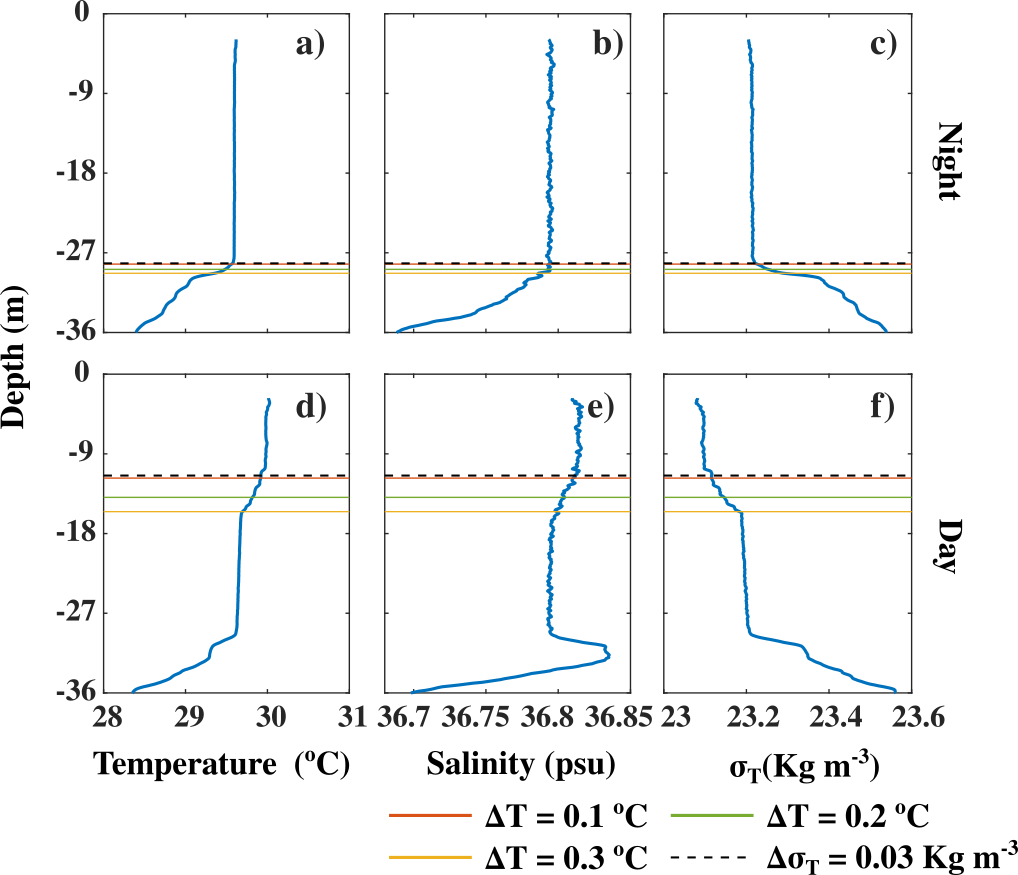


Figure S7. Same as Figure S6; but for the day 14 May 2019.


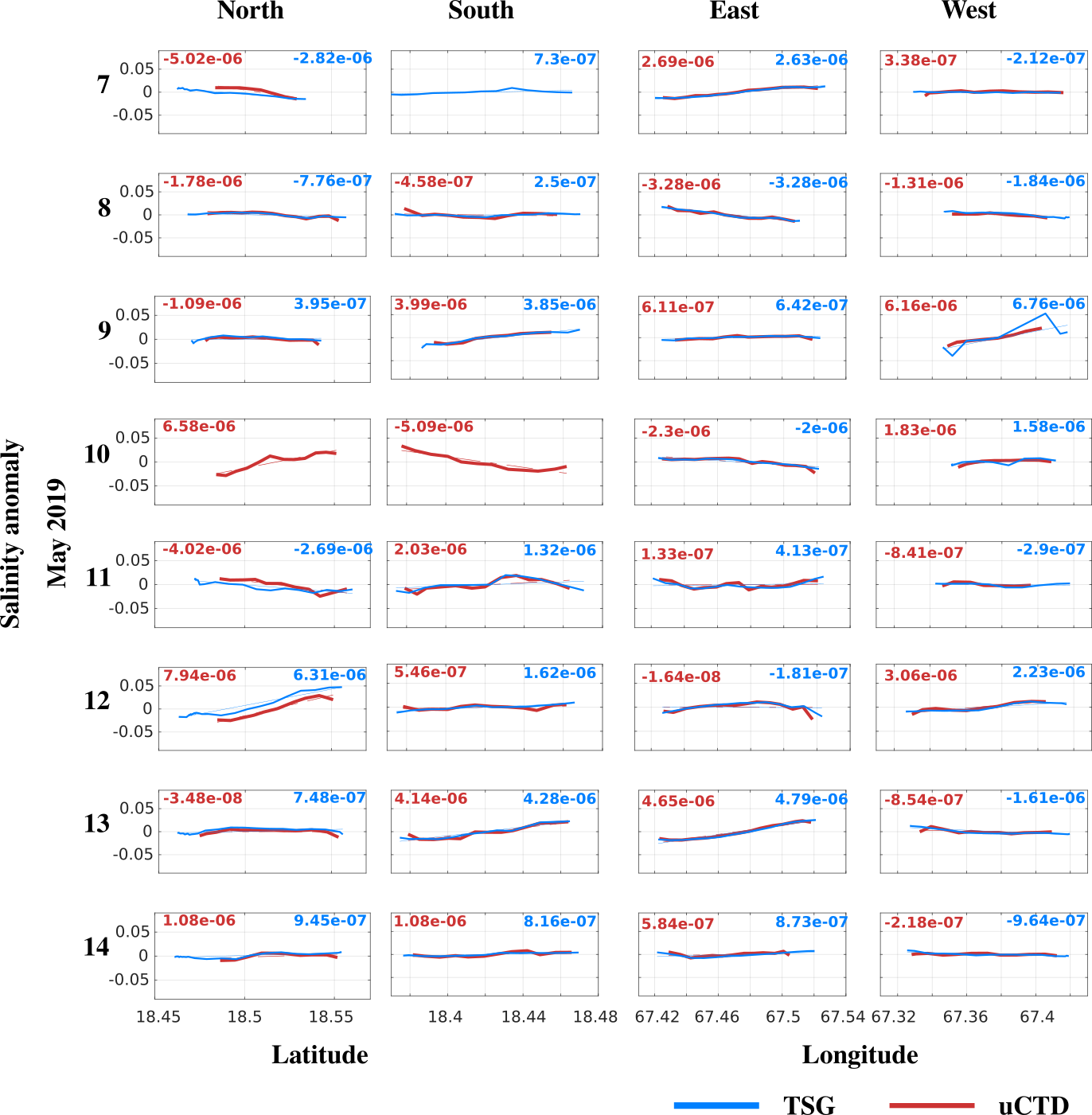


Figure S8 (a). The salinity anomaly (the zonal or meridional average of salinity from the respective individual transect is removed from each data point) along the meridional ((a) north to the centre and (b) south to centre) and zonal ((c) east to centre and (d) west to centre) estimated from the TSG (thick blue lines) and uCTD (thick red lines) transects at a depth of 6 m along with the corresponding linear regression lines (dashed blue (TSG) and red (uCTD) lines) during 7-14 May 2019 (S19). The slope of the regression lines estimated from TSG and uCTD are written in the individual panels (psu day^-1^) in the blue and red font, respectively. The number written in bold black font near each panel in the first column was represented when the observation was conducted between 7-14 May 2019.


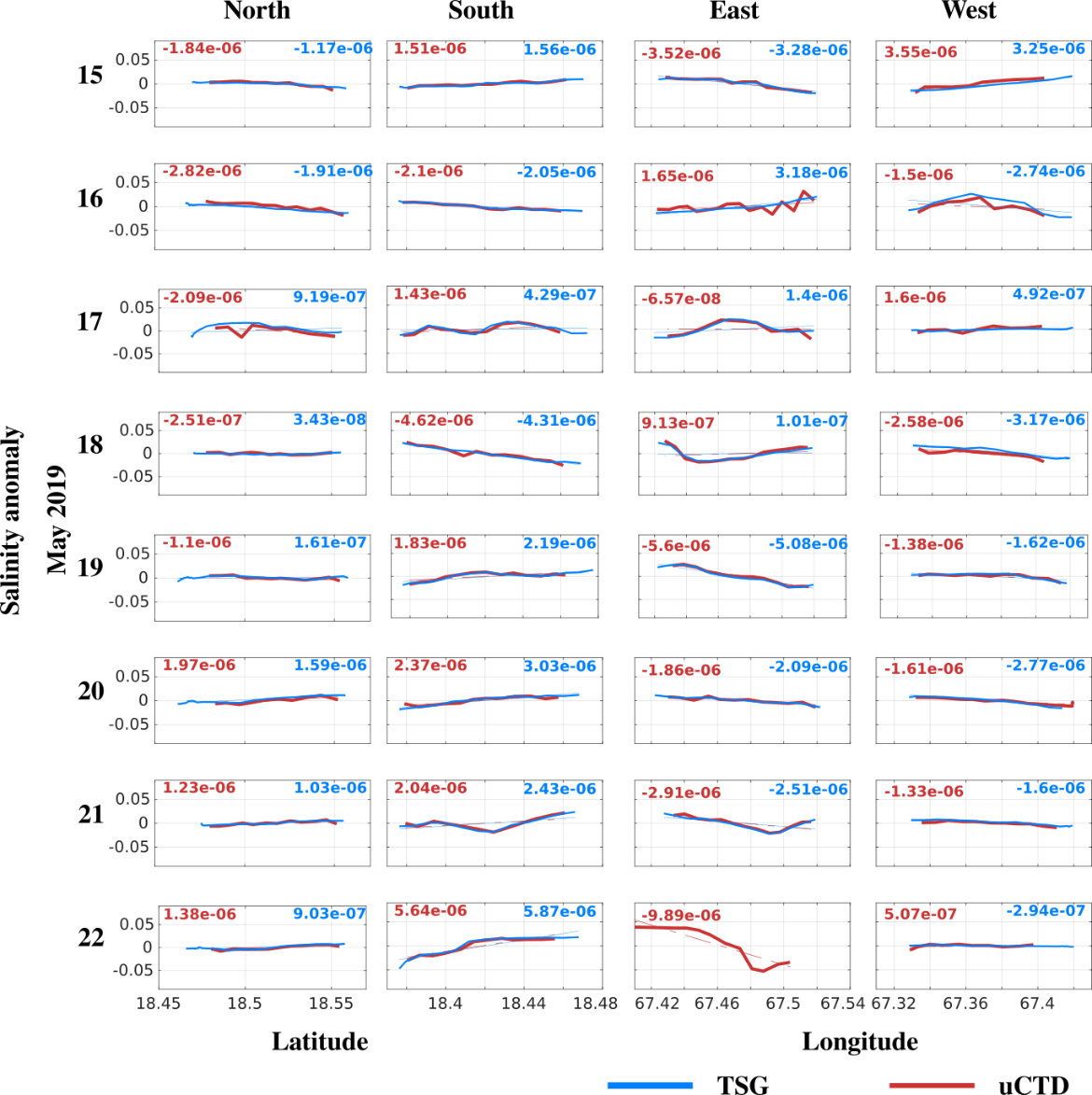


Figure S8 (b). Continuation of Figure S8a, but for during 15-22 May 2019


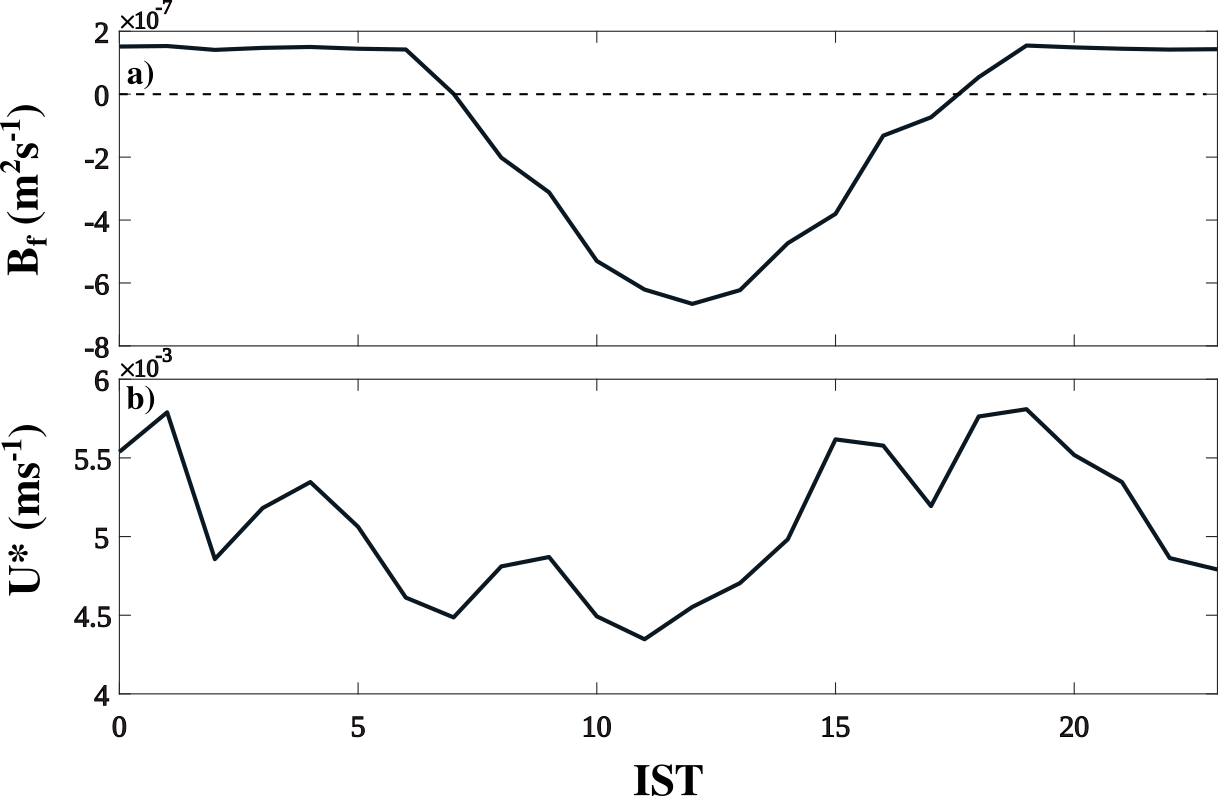


Figure S9. The composite of sub-daily evolution of (a) buoyance flux (B_f_x10^-7^; m^2^ s^-1^), and (b) frictional velocity (U^*^, ms^-1^). Time in IST hours.

### **S1. Selection of a threshold value for the mixed layer depth.**

Different criteria, based on difference (*∆*) or gradients (∂/∂z) in temperature and density, are discussed in the previous studies to estimate the mixed layer depth (Table-1 of de Boyer Montégut 2004). However, the study by *Schneider and Müller* [1990] and *Pujiana et al.* [2018] used a criterion such as a density increase (*∆σ_t_*) of magnitude 0.01 kg m^-3^ or 0.03 kg m^-3^ above the surface value in order to resolve the diurnal variability of mixed layer depth. Our primary interest here is to demarcate the diurnal thermocline (DT) region, a transition layer between the base of the warm daytime mixed layer and the nocturnal mixed layer. However, our analysis suggested that the mixed layer depth estimated using the temperature criterion (*∆T*=0.1°C) coincides approximately with subjective estimates of mixed layer depth during the afternoon and night (Figures S6 and S7). A value of 0.2°C for *∆T* yielded a slightly deeper mixed layer, such as the estimated mixed layer depth was in the main thermocline during the nighttime and within the diurnal thermocline during the afternoon (Figures S6 and S7). We also compared the mixed layer depth estimation using temperature criterion (*∆T*) of the magnitude of 0.1°C with the estimation based on density criterion (*∆σ_t_*) of magnitude 0.03 kg m^-3^. The latter criterion is consistent with density change equivalent to a temperature change of magnitude of 0.1 °C in the absence of no salinity variability in the mixed layer. It is found that the mixed layer depth estimated using temperature criterion (*∆T*) of the magnitude of 0.1°C with density criterion (*∆σ_t_*) of magnitude 0.03 kg m^-3^ shows reasonably good agreement with a correlation of 0.97 and a root mean square difference (RMSD) of 0.2 m. These differences are relatively small compared to the standard deviation (7.3 m) of mixed layer depth estimated using the temperature criterion (*ΔT*) criterion of magnitude 0.1°C and density criterion (*∆σ_t_*) of magnitude 0.03 kg m^-3^. Hence, we used a temperature decrease (*∆T*) of magnitude 0.1 °C with respect to surface value to estimate mixed layer depth. For the mixed layer salinity budget, a depth was estimated where the temperature is 0.3°C lower than the surface value, and this criterion reasonably captured the base of the diurnal thermocline.

**Reference**

de Boyer Montégut, C., Madec, G., Fischer, A. S., Lazar, A. & Iudicone, D. Mixed layer depth over the global ocean: An examination of profile data and a profile-based climatology. *J. Geophys. Res.,* 109, C12003, (2004).

Schneider, N. & P. Müller, P. The meridional and seasonal structures of the mixed layer depth and its diurnal amplitude observed during the Hawaii-to-Tahiti shuttle experiment. *J. Phys. Oceanogr.,* 20, 1395–1404 (1990).

Pujiana, K., Moum, J. N. & Smyth, W. D. The Role of Turbulence in Redistributing Upper-Ocean Heat, Freshwater, and Momentum in Response to the MJO in the Equatorial Indian Ocean. *J. Phys. Oceanogr.*, 48(1), 197-220. (2018)
